# Hotspots in the genomic architecture of field drought responses in wheat as breeding targets

**DOI:** 10.1007/s10142-018-0639-3

**Published:** 2018-11-16

**Authors:** Sergio Gálvez, Rosa Mérida-García, Carlos Camino, Philippa Borrill, Michael Abrouk, Ricardo H. Ramírez-González, Sezgi Biyiklioglu, Francisco Amil-Ruiz, The IWGSC, Gabriel Dorado, Hikmet Budak, Victoria Gonzalez-Dugo, Pablo J. Zarco-Tejada, Rudi Appels, Cristobal Uauy, Pilar Hernandez

**Affiliations:** 1 Departamento de Lenguajes y Ciencias de la Computación, ETSI Informática, Campus de Teatinos, Universidad de Málaga, 29071 Málaga, Spain; 2 Instituto de Agricultura Sostenible (IAS), Consejo Superior de Investigaciones Científicas (CSIC), Alameda del Obispo s/n, 14004 Córdoba, Spain; 3 John Innes Centre, Norwich Research Park, Norwich, NR4 7UH UK; 4 Institute of Experimental Botany, Centre of Plant Structural and Functional Genomics, CZ-78371 Olomouc, Czech Republic; 5 Biological and Environmental Science & Engineering Division, King Abdullah University of Science and Technology, Thuwal, 23955-6900 Kingdom of Saudi Arabia; 6 Department of Plant Sciences and Plant Pathology, Montana State University, Bozeman, MT 59717-3150 USA; 7 Bioinformatics Unit, SCAI, Campus Rabanales, University of Córdoba, 14014 Córdoba, Spain; 8 International Wheat Genome Sequencing Consortium, 2841 NE Marywood Ct, Lee’s Summit, MO 64086 USA; 9 Departamento de Bioquímica y Biología Molecular, Campus de Excelencia Internacional Agroalimentario (ceiA3), Universidad de Córdoba, Campus Rabanales C6-1-E17, 14071 Córdoba, Spain; 10 Veterinary and Agricultural Sciences, University of Melbourne, Gratten St, Parkville, Victoria 3010 Australia; 11 Department of Economic Development, AgriBio, Centre for AgriBioscience, Jobs, Transport and Resources, La Trobe University, 5 Ring Rd, Bundoora, Victoria 3083 Australia

**Keywords:** Transcriptomics, Plant spectral traits, Field drought tolerance

## Abstract

Wheat can adapt to most agricultural conditions across temperate regions. This success is the result of phenotypic plasticity conferred by a large and complex genome composed of three homoeologous genomes (A, B, and D). Although drought is a major cause of yield and quality loss in wheat, the adaptive mechanisms and gene networks underlying drought responses in the field remain largely unknown. Here, we addressed this by utilizing an interdisciplinary approach involving field water status phenotyping, sampling, and gene expression analyses. Overall, changes at the transcriptional level were reflected in plant spectral traits amenable to field-level physiological measurements, although changes in photosynthesis-related pathways were found likely to be under more complex post-transcriptional control. Examining homoeologous genes with a 1:1:1 relationship across the A, B, and D genomes (triads), we revealed a complex genomic architecture for drought responses under field conditions, involving gene homoeolog specialization, multiple gene clusters, gene families, miRNAs, and transcription factors coordinating these responses. Our results provide a new focus for genomics-assisted breeding of drought-tolerant wheat cultivars.

**Electronic supplementary material:**

The online version of this article (10.1007/s10142-018-0639-3) contains supplementary material, which is available to authorized users.

## Introduction

Maintaining plant growth and yield under drought stress is a major objective for wheat breeding programs worldwide. At the molecular level, drought response is a complex genetic mechanism (McWilliam 1989) involving multiple genes, transcription factors, miRNAs, hormones, proteins, co-factors, ions, and metabolites (Budak et al. 2015). The complexity of this trait has hindered efforts to advance breeding for drought using conventional DNA-based genetic markers. Research efforts have therefore been devoted to the integration of physiology attributes to derive novel diagnostic markers and facilitate the selection of parents and further breeding activity. These efforts include the translation of physiological research to the field by implementing physiologically defined traits within breeding programs (Reynolds et al. 2012) and more recently high-throughput proxies have used spectral traits of plants to aid in germplasm selection (Condorelli et al. 2018; Gonzalez-Dugo et al. 2015).

Moving forward, a prerequisite for genomics-informed cultivar improvement will be the understanding of the genetic and genomic basis of the drought tolerance mechanisms. These studies have lagged behind in wheat compared to other species primarily due to its large (1C = 16 Gb), hexaploid, and complex genome, with over 85% repetitive DNA content. The recent fully annotated and mapped reference sequence for the bread wheat cultivar “Chinese Spring” (IWGSC 2018) provides the basis for improved analyses with the potential to derive novel breeding targets. Among these, transcriptomics is a powerful approach to identify gene regulatory networks, transcription factors, and miRNAs involved in the stress response and to identify differentially expressed genes during drought treatments.

Traditionally, transcriptomics analyses of drought stress have been carried out under highly controlled environmental conditions by withholding water or using an osmotic stress agent such as polyethylene glycol (PEG) or mannitol, thereby leading to progressive drought stress (Li et al. 2017; Liu et al. 2015; Qiu et al. 2017). Under agronomic conditions, however, plants are often exposed to milder, non-sustained drought stress (Fleury et al. 2010; Ma et al. 2017; Passioura 2007), particularly under rainfed conditions, which accounts for over 70% of the wheat crop worldwide (You et al. 2014). Integrating the water status analysis in the field makes the analysis of molecular drought responses under real agronomic water-limited conditions feasible.

In this work, we used an interdisciplinary approach to investigate the genomic architecture of drought stress under field agronomic conditions, by combining state-of-the-art monitoring of plant spectral traits related to water and nutrient status under field conditions with gene expression analysis.

## Results and discussion

**Fig. 1:**
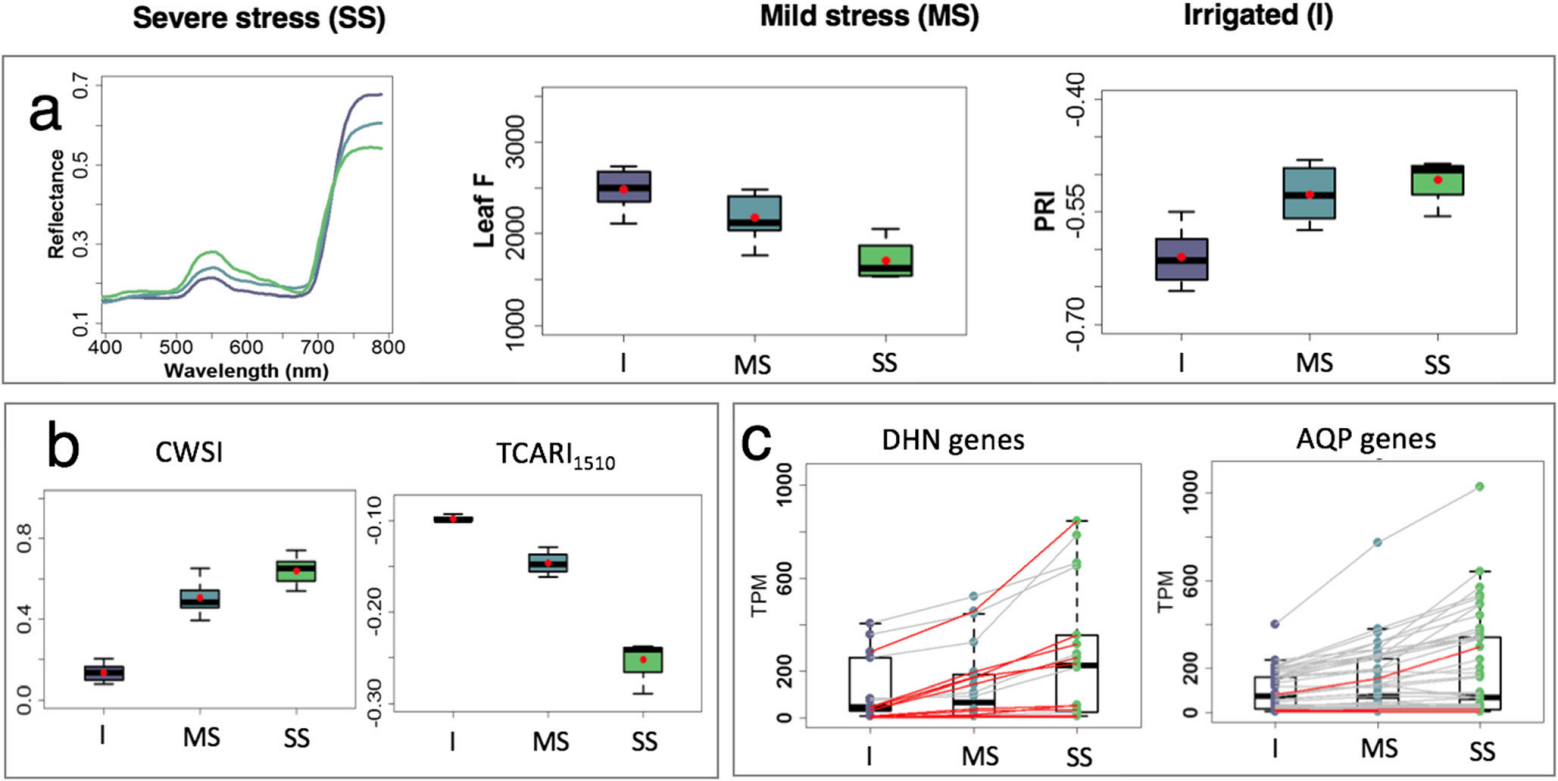
Integrated phenotyping workflow at the farm plot and flag leaf tissue levels and gene expression analysis for dehydrin and aquaporin gene families. **a** Remote sensing data thermal imagery of farm sites (**a**) reflectance, leaf fluorescence (F), and Photochemical Reflectance Index (PRI) measured at the flag leaf for severe stress (SS), mild stress (MS), and irrigated (I) samples. **b** Crop Water Stress Index (CWSI) and Transformed Chlorophyll Absorption in Reflectance Index (TCARI_1510_) remote sensing indexes measured at the canopy level. **c** Expression values (transcripts per million, TPM) of dehydrin (DHN) and aquaporin (AQP) genes in tissues at the three water levels. The values of individual genes are represented by boxplots and connected along the three water levels. Differentially expressed genes are connected by red lines. Boxplots include the median and 25–75 interquartile range. Means in (E) are represented by red dots. Color codes used in the figure correspond to the irrigated (I, purple), mild stress (MS, turquoise), and severe stress (SS) watering status analyzed

We grew standard-scale breeding plots of the reference wheat cultivar “Chinese Spring” to assess the effects of varying levels of drought stress and to evaluate this crop in a canopy context under field conditions in the temperate Mediterranean climate of Southern Spain (Fig. S1). We established three different watering regimes and used plant spectral traits derived from high-resolution hyperspectral and thermal imagery (Zarco-Tejada et al. 2018) in conjunction with physiological water status analysis (Fig. 1a) to determine the severity of the stress. These watering regimes resulted in severe drought stress, intermediate/mild drought stress, and a non-stressed, fully irrigated control condition, as quantified by spectral analyses. Plants under severe drought stress had lower stomatal conductance, CO_2_ assimilation, fluorescence emission, and photosynthetic rates compared to their counterparts under mild drought stress and irrigation. Focusing on the mid-grain filling stage (Zadok’s growth stage Z77, Zadoks et al. 1974), the plants showed significant differences in leaf reflectance, fluorescence, and the xanthophyll-sensitive Photochemical Reflectance Index (PRI; Fig. 1a, Table S1; Gamon et al. 1992). Crop water and nitrogen status were also significantly altered by the treatments, as detected by the spectral plant functional traits Crop Water Stress Index (CWSI, Gonzalez-Dugo et al. 2015) and Transformed Chlorophyll Absorption in Reflectance Index (TCARI_1510_; Fig. 1b, Table S1). These results confirmed that the treatments were successful in imposing a stepwise change in stress conditions.

**Fig. 2:**
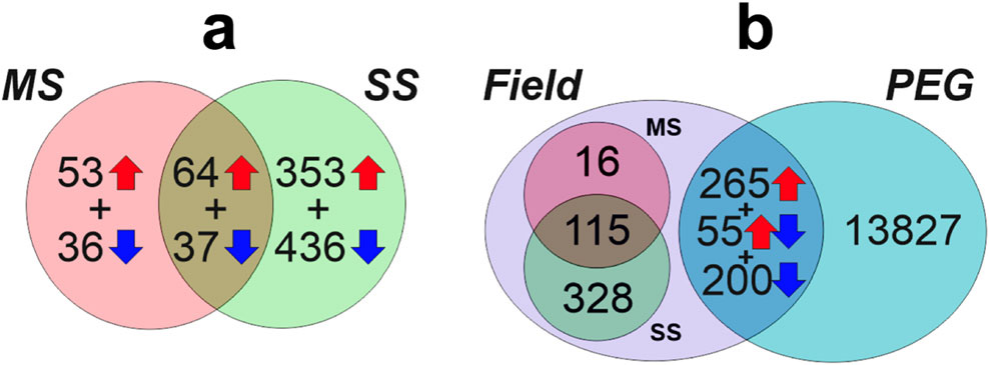
Summary of common and specific DE gene responses. **a** DE genes are grouped by field stress level and classified into up- and downregulated. One hundred and one genes (53 upregulated + 37 downregulated) are differentially expressed at both stress levels imposed (mild stress MS, severe stress SS), and **b** comparison between field drought in flag leaf (this study) and PEG shock in seedling leaves (Liu et al. 2015). Genes DE in field are grouped for the two stress levels; in total 459 (16 + 115 + 328), DE genes were unique to field drought stress. The number of genes uniquely responsive to drought stress in the field ranged from 131 (16 + 115 for mild stress) to 443 (115 + 328 for severe stress) common DE genes among studies (520 in total, 265 + 55 + 200) are classified into up-, down-, and opposite-regulated gene groups (regulated in different directions in each experiment)

We determined the gene expression patterns and drought responses of this field-grown wheat by performing RNA sequencing (RNA-Seq) of the uppermost (flag) leaf from three replicates for the three treatments at mid-grain filling (Z77). We performed differential expression analysis of the RNA-Seq data using two complementary bioinformatics pipelines (STAR/DESeq and Kallisto/Sleuth) and defined statistical thresholds that account for the higher inter-replicate variability obtained under field conditions compared to controlled environments (Fig. S2). Using a consensus and stringent threshold across the two pipelines (|lg_2_FC, β| > 1.0 and *p*-adjusted *Q* value < 0.05), we identified 979 drought-responsive differentially expressed (DE) genes, including 470 that were upregulated and 509 that were downregulated between either stress condition and the irrigated control. Only 10.3% of genes (101 genes) exhibited differential expression at both stress levels (severe and mild stress, Fig. S2a). We compared these field drought stress-responsive DE genes with the results of de novo analysis of publicly available “drought” RNA-Seq datasets from PEG shock-stressed seedlings (Liu et al. 2015). We identified 459 DE genes that were unique to field drought stress (46.9%), whereas 520 genes (53.1%) were shared with PEG shock stress (Fig. 2b). Of the 520 genes shared among experiments, 10.6% (55 genes) exhibited opposite differential expression patterns when comparing experiments. Some of these differences likely stem from the different wheat cultivars and developmental stages examined. However, these results suggest that plants grown under agronomic field conditions respond to drought stress in ways that cannot be replicated in controlled conditions. This would argue that field experiments will be required to elucidate gene networks and pathways of relevance to breeding for drought tolerance (Fig. 2).

Using the physical positions of genes across chromosomes, we found an enrichment of field drought-responsive DE genes in the distal regions of chromosomes compared to centromeric intervals (Tables S2–S3, Fig. S3). Only 3.4% of DE genes were found in centromeric regions compared to the expected 10% (Table S2) (IWGSC 2018). This distribution pattern is consistent with the enrichment of stress-related genes at the distal ends of chromosomes compared to proximal regions in the wheat genome (Ramírez-González et al. 2018), providing evidence that this altered distribution pattern is functionally relevant during field drought stress responses.

Several genes encoding enzymes in the carotenoid biosynthesis pathway were overexpressed under drought stress (Fig. S4). Specifically, genes involved in the transformation of beta-carotene to zeaxanthin (*Z*) and the transformation of violaxanthin (*V*) to xanthoxin were upregulated under drought stress, as were genes involved in ABA biosynthesis (Fig. S4a). We measured several plant spectral traits to detect the changes in absorption of *V*, antheraxanthin (*A*), and *Z*, indicating xanthophyll epoxidation [epoxidation state (EPS) = (*V* + 0.5 *A*) ∕ (*V* + *A* + *Z*)]. The changes in expression of drought-responsive genes likely led to an increase in zeaxanthin biosynthesis and the degradation of violaxanthin. These traits were detected by spectral indicators due to their link to carotenoid absorption, which was reflected by a decrease in leaf and canopy reflectance between 515 and 570 nm (CAR, Fig. S4b) in response to drought stress. Field drought repressed the expression of several glutamine synthetase genes, especially the *GS1c-D* homoeolog (Table S6), with decreased expression of this gene coinciding with decreasing TCARI_1510_ values and N contents. Therefore, for carotenoid and nitrogen pathways, changes at the transcriptional level were reflected in the physiological data obtained from spectral instruments in the field.

For other photosynthesis-related functional traits, drought treatments resulted in a decrease in the solar-induced fluorescence emission (SIF, Guanter et al. 2014; Fig. S5). However, changes at the transcriptional level were observed only for a single component of photosystem II, *PsbQ*, which was overexpressed in response to drought (Table S4). These results suggest that drought-induced changes in pathways related to photosynthesis are likely under more complex post-transcriptional control than those described above (Fankhauser and Aubry 2017). Consistent with this, the miRNA repertoires differed among treatments (Fig. S6, Table S5–S6). Severe drought-stressed plants had significant over-representation of miRNA-target genes related to gene ontology terms such as hydroquinone: oxygen oxidoreductase activity (GO:0052716) and response to water deprivation (GO:0009414) (*padj <* 0.001; Table S7), which is consistent with the spectral analysis and RNA-Seq results.

**Fig. 3:**
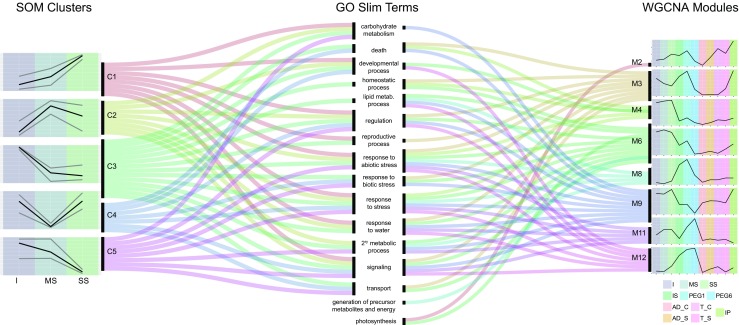
Alluvial plots showing common gene ontology enrichment terms of all SOM clusters (left) and stress response-significant WGCNA modules (right). Black lines in SOM analysis (left) correspond to scaled mean TPM values, gray lines correspond to maximum and minimum values. The first three columns of the WGCNA modules (left to right) correspond to irrigated (I), mild stress (MS), and severe stress (SS) phenotypes. Black lines correspond to mean VST (TPM) values. Coded phenotypes (Table S9): IS seedling PEG shock control, PEG1 seedling 1-h PEG stress, PEG 6 seedling 6-h PEG stress, AD_C anther stage irrigated shelter phenotype, AD_S anther stage drought-stressed shelter phenotype, T_C tetrad stage irrigated shelter phenotype, T_S tetrad stage drought-stressed shelter phenotype, IP non-stressed pot phenotypes

We extended our initial analysis of DE genes and examined the co-expression of 3678 drought-responsive genes (adjusted *p* value *<* 0.05 in at least one of the two pipelines) by clustering them using self-organizing maps (SOM, Fig. S7a) (Törönen et al. 1999). Co-expression modules 1 and 3 were notable in that they showed a pattern of up- and downregulation, respectively, with increasing stress (Fig. 3). These modules also had the highest number of significantly enriched GO terms (Fig. S8), in categories such as regulation of gene expression, signaling, and response to water stress (module 3; Table S8). For the gene homoeolog triads, we found that while most (57.7%) were assigned to the same co-expression module, there were clear homoeolog-specific drought responses (Fig. S7b). This is consistent with the overall (whole genome) homoeolog expression distribution across the three watering status conditions analyzed (Fig. S9). Extending the co-expression analysis to a wider set of 30,180 genes (*p* value < 0.1) and publicly available drought experiment RNA-Seq data (www.wheat-expression.com, Table S9), we performed a weighted gene correlation network analysis (WGCNA) (Langfelder and Horvath 2008) and detected specific modules enriched for response to water, regulation, and signaling GO terms (Fig. 3).

**Fig. 4:**
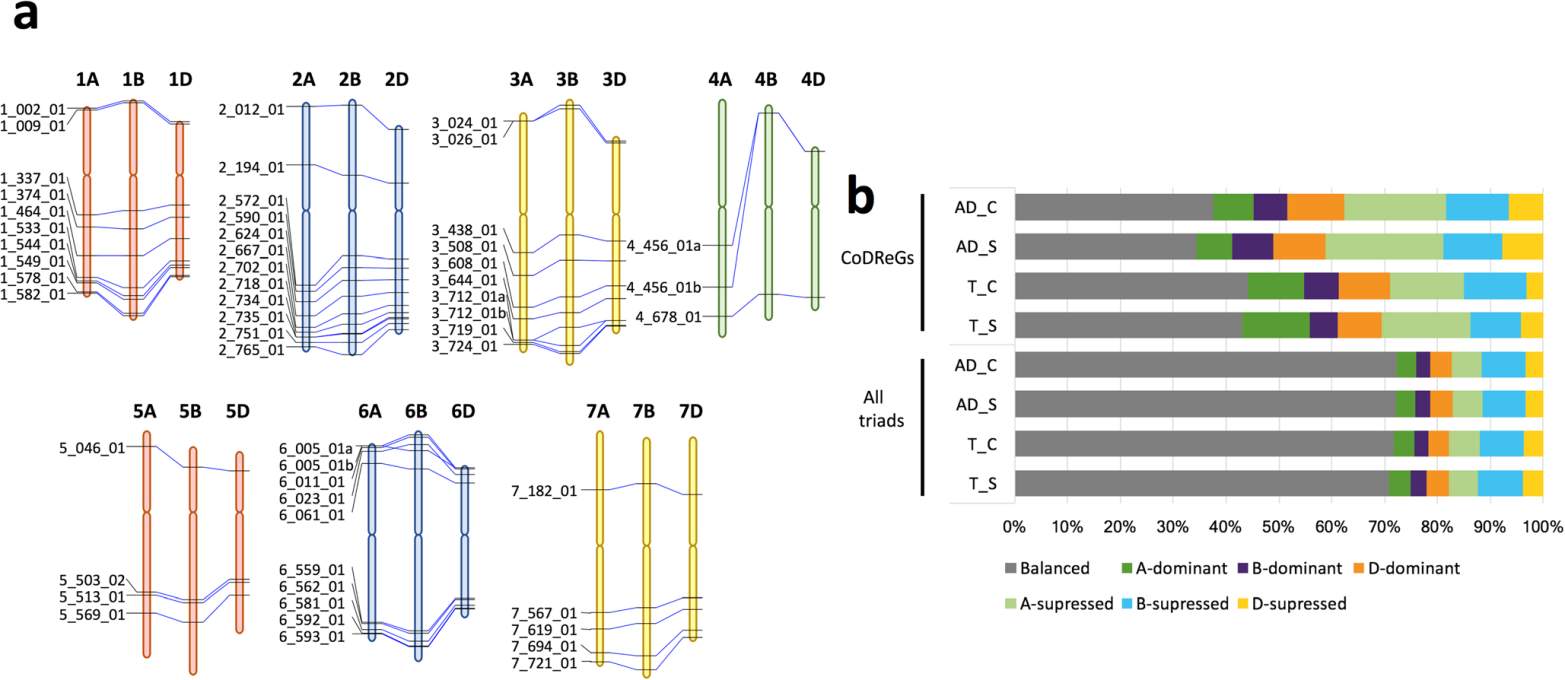
Clusters of drought-responsive genes (CoDReGs). **a** Physical location on the RefSeqv1 chromosome pseudomolecules. **b** Relative homoeolog gene expression (corresponding to the seven classes categories described in Fig. S9) across the genome and CoDReGs, for the three stress levels analyzed: irrigated (I), mild stress (MS), and severe stress (SS)

We uncovered a genomic architecture for drought responses that included the existence of genomic regions hosting compact clusters of genes that were differentially expressed under drought stress conditions, which we denominated clusters of drought-responsive genes (CoDReGs). In analogy to Anhydrobiosis-related gene islands (ARIds) and cluster of desiccation-associated genes (CoDAGs) (Costa et al. 2017), for a given gene cluster to be considered a CoDReG, a set of at least three genes must (i) be located within a 1-Mb region, (ii) belong to an ABD homoeolog triad, and (iii) include at least one gene that was differentially expressed in field drought stress conditions. The 51 CoDReGs (Fig. 4a) included 915 genes from four of the five SOM modules (Table S10) and were preferentially located in distal parts of the wheat chromosomes, consistent with our previous results for DE genes. Not all genes within a CoDReG belonged to the same SOM module, as they sometimes showed distinct expression patterns in response to field drought stress. Genes encoding proteins involved in a range of biological processes formed CoDReGs (Table S10), e.g., dehydrin (*DHN*) genes and genes involved in carbohydrate and lipid metabolism. Over half of wheat CoDReGs (55%) were in regions syntenic to rice drought quantitative trait loci (QTL) (Fig. S10, Tables S11-S12), which could be due to conserved drought response mechanisms between wheat and rice. It will be interesting to explore whether the wheat-specific CoDReGs might underlie distinct drought tolerance mechanisms in wheat that stem from its more temperate growing environment.

To investigate the coordination in the relative expression of the three wheat subgenomes in response to drought stress, we analyzed homoeolog expression bias (Ramírez-González et al. 2018) in the 19,887 wheat genome triads and CoDReGs (Fig. 4b). Across all expressed triads, ~ 65% showed balanced expression between the A, B, and D subgenomes, independent of the drought treatment (Fig. S9). However, CoDREGs had higher homoeolog expression bias, with only ~ 30% of triads showing balanced expression and 70% of triads having higher or lower expression from a single homoeolog with respect to the other two (Fig. 2b, Table S13). This expression bias in CoDReGs became more severe as the drought stress level increased (Fig. 2b) suggesting that homoeolog-specific expression of CoDReGs plays a role in the stress response. We independently validated these findings on homoeolog specialization of gene expression in1 CoDREG regions (Fig. S11) using the additional wheat drought experiments available at www.wheat-expression.com (Borrill et al. 2016; Ramírez-González et al. 2018).

**Fig. 5:**
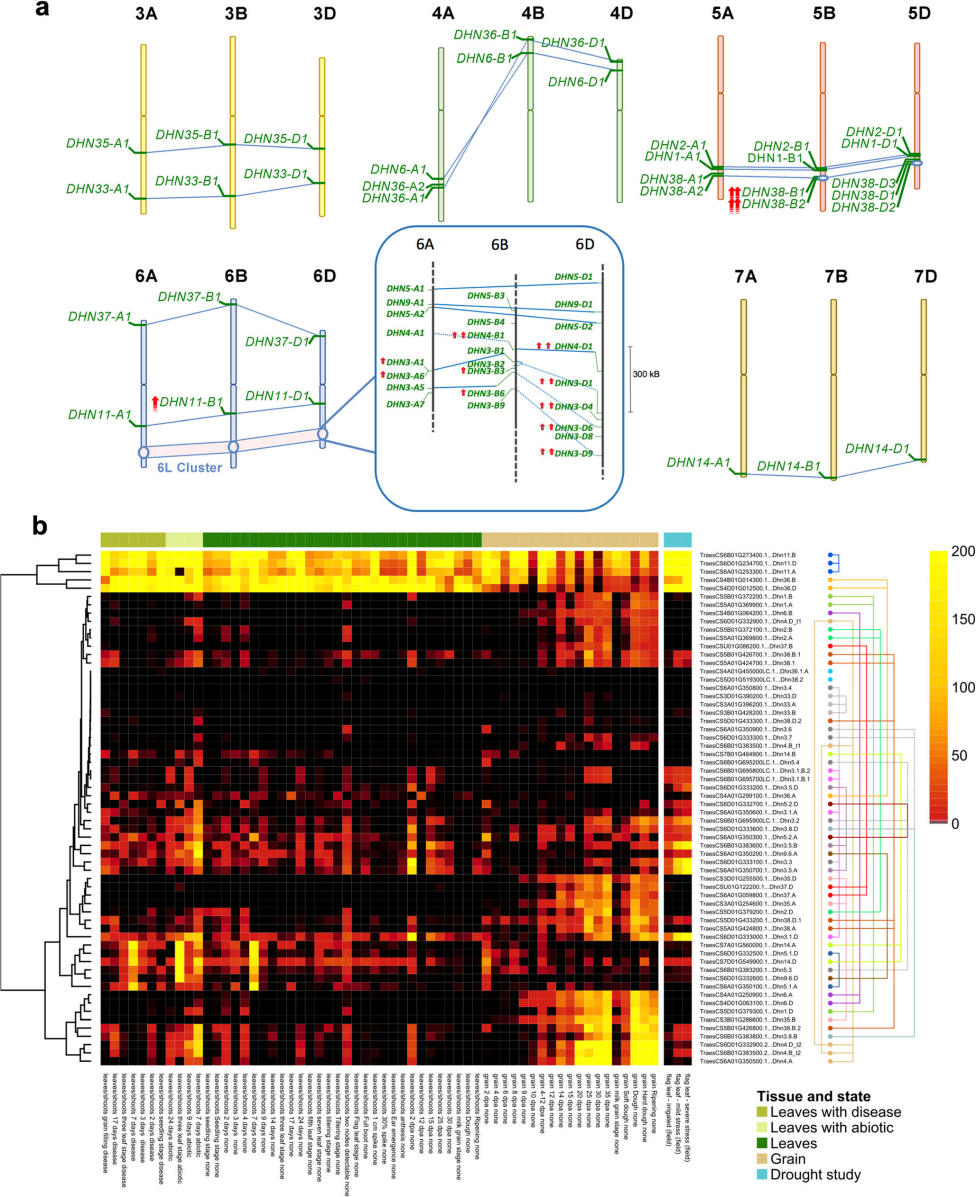
Analysis of the dehydrin (*DHN*) gene family. **a** Chromosomal positions of the 60 annotated *DHN* genes. The dehydrins preceded by red arrows were differentially expressed (lgFCvalue > 1, *p*-adjusted value < 0.05) in the severe stressed condition (one arrow) or in both the mild stress and the severe stress conditions (two arrows). The 6L cluster containing 25 *DHN* genes (CoDReG_6_581_01) is expanded. Blue lines connect homoeologous genes, and dotted blue lines connect genes with an unassigned phylogenomic relationship. 6B dehydrin genes had the highest expression levels. **b** Clustering of *DHN* genes based on their expression patterns across all available experiments at www.wheat-expression.com (clustering on the left) and homoeolog analysis (clustering on the right). Columns indicate low-level tissue grouping; the three columns on the right represent the samples examined in this study. Colored lines (right) connect homoeologs

The dehydrin gene family was of particular interest given its known role in the drought response (Close 1996) and the differential expression of these genes in our RNA-Seq analyses. We manually annotated dehydrin genes and analyzed their structures based on dehydrin typical conserved sequences motifs (Malik et al. 2017) (K, Y, and S) and named the 60 resulting genes (Fig. 5a, Fig. S12, Table S14). We found an average of 1.97 K-motifs, 0.87 Y-motifs, and 0.73 S-motifs per dehydrin in the wheat genome. The dehydrin genes were clustered on chromosomes 5 and 6 (37 of 50 mapped), with 28 dehydrin genes (46.7%) located on the long arm of group 6 chromosomes (6L). Thirteen dehydrin genes were overexpressed in the flag leaf in response to field drought (Fig. 5a), 11 of which were located on 6L (Fig. 5a), with the remaining two on 5BL (Fig. 5a). This result is in contrast with other drought-related gene families, such as aquaporins (Fig. 1c), which were not significantly enriched in distal ends of chromosomes and had few DE members under drought stress (8/162; Table S3). The main 6L dehydrin cluster (Fig. 5a) corresponded to CoDReG_6_581_01, which is in the same physical position as previously defined wheat drought Meta-QTLs on chromosomes 6B and 6D (MQTL53 and MQTL56 (Acuña-Galindo et al. 2015)). The identification of this region across independent studies highlights its potential as a breeding target.

The close physical proximity of the chromosome 5 and 6 *DHN* genes, together with the phylogenetic analysis, suggests that these clusters arose through multiple rounds of duplications. This is reminiscent of other major stress tolerance loci, such as the *CBF* cluster associated with cold tolerance in wheat. In the *CBF* cluster, copy number variation determines the level of cold resistance (Knox et al. 2010), similar to other major adaptation loci in wheat such as photoperiod response (*PPD-1*) and vernalization (*VRN-1*, Díaz et al. 2012; Würschum et al. 2015; Zhu et al. 2014). It will be important to determine haplotypes across these two *DHN* clusters and define the effect of copy number variation on drought tolerance.

We examined the expression of all dehydrin genes based on 850 available RNA-Seq datasets (Ramírez-González et al. 2018) and clustered these data alongside our field drought results (Fig. 5b, Fig. S13). We identified both common and different DE dehydrin genes in response to field drought vs. PEG-induced drought stress (Fig. 2). Interestingly, some drought-responsive dehydrin genes, such as *DHN38*, were grain-specific in the absence of stress, suggesting that a common mechanism functions between abiotic stress and grain dehydration. According to our synteny analysis, only two homoeolog triads (*DHN11* and *DHN35*) were syntenic with rice and *Brachypodium* (Table S15). This together with the physical clustering indicates a distinct pattern of translocations and expansion of this family in wheat (IWGSC 2018). The *DHN11* triad was expressed across most tissues, with the B homoeolog differentially expressed under field drought conditions and syntenic with a rice drought QTL for osmotic adjustment (Zhang et al. 2001) (Fig. S14). By contrast, other highly drought-responsive genes identified in our study, like *DHN3-A1* and *DHN3-D6* (Fig. 5b), were not syntenic with rice genes. These results support the presence of common and species-specific responses, as indicated by CoDReG analysis. Interestingly, the homoeologous *DHN4-B1* and *DHN4-D1* genes expressed alternative splicing variants with different responses to drought stress (Fig. 5b), highlighting the potential for the specialization of homoeologs.

We identified 228 TFs that were predicted to regulate dehydrin genes (Table S16) based on a Genie3 network (Ramírez-González et al. 2018). The AP2/EREBP and NAC TF families were most frequently predicted to regulate dehydrin genes (16.7% and 13.6% of all predicted TFs, respectively; Table S16), and these families were enriched for dehydrin targets compared to all downstream target genes (*χ*^2^, *p* value *<* 0.001, Table S16). Many rice and Arabidopsis orthologs of these AP2/EREBP and NAC TFs have been shown to regulate drought responses in these species (Table S17; Joshi et al. 2016). Furthermore, one ortholog, *OsDREB1A*, directly binds to the promoter of *OsDHN* (Lee et al. 2013), a rice dehydrin gene, suggesting a conserved mechanism regulating dehydrin gene expression between rice and wheat. The AP2/EREBP TFs were predicted to target 49.7% of dehydrins across all tissues (Fig. S15), increasing to 77.8% in the ripening grain, suggesting a potential regulatory mechanism for the tissue-specific expression of dehydrin genes in ripening grain mediated by a subset of AP2/EREBP TFs (Fig. S16).

**Fig. 6:**
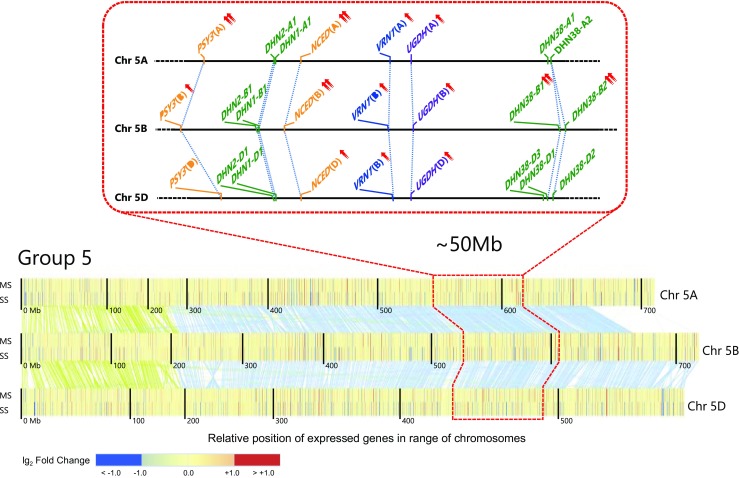
Transcriptional changes affecting several drought-responsive genes within a 50-Mb genomic region on the long arm of group 5 chromosomes. Differential expression under field drought of genes on group 5 chromosomes (below) and enlarged drawing of the locations of genes in the carotenoid pathway (*PSY3*, *NCED*), as well as *dehydrin* (*DHN*), *VRN1*, and *UDP glucose-6-dehydrogenase* (*UGDH*). Each chromosome is represented by two bands of vertical lines associated with mild stress (upper band) and severe stress (lower band). Vertical lines correspond to individual genes with the color indicating expression with respect to the irrigated control. Green and blue lines connect homoeologs on the short and long arms of the chromosome, respectively

As part of defining the genome architecture of field drought response, we also identified a ~ 50-Mb genomic region on the long arm of group 5 chromosomes containing several drought-responsive DE genes (Fig. 6) and genes previously associated with drought tolerance (Quarrie et al. 1994). This region included genes known to affect drought tolerance (Close 1996; Iuchi et al. 2001) including those from the carotenoid pathway (*PSY3, NCED*) and the *DHN* chromosome 5 cluster, as well as a gene encoding UDP glucose-6-dehydrogenase (Table S5), which partitions carbon resources during spike development to ensure fertility and grain yield (Ferreira and Sonnewald 2012). This interval also contained the major vernalization gene *VRN1* (Yan et al. 2003), which was recently shown also to affect root system architecture in wheat (Voss-Fels et al. 2017). Given the strong selection pressure on *VRN1* within breeding programs, it will be important to determine the extent of linkage drag across this 50 Mb region to help define the most locally relevant haplotypes, combining appropriate vernalization requirements with drought tolerance, for use in marker-assisted breeding.

In summary, we applied an interdisciplinary approach that combined water status field phenotyping information with gene expression data. We identified commonalities between spectral trait alterations quantified from specific narrow bands and transcriptional changes in leaves. This approach defined genomic regions that influence field drought responses in polyploid wheat. Extending this approach to other plant functional traits for precise field phenotyping will facilitate the discovery of novel and valuable expressed alleles for breeding. Several of the identified regions overlapped with rice QTLs, while other regions overlapped exclusively with wheat QTLs, providing a strategy to prioritize breeding targets in wheat. This work, alongside the new genomic resources in wheat, provides a focus for breeding of drought-tolerant wheat cultivars by exploiting the genome architecture of gene clusters and the opportunity to adjust the interactions within gene networks through genomics-informed breeding.

## Experimental methods

### Field sampling, physiological data collection, and hyperspectral and thermal image data collection and processing

The field experiment was carried out using the hexaploid wheat cultivar *Triticum aestivum* cv. “Chinese Spring,” following standard wheat agronomical practices in the South of Spain (Seville province) at two different locations, Ecija (37° 32′ 17″ N, 5° 06′ 57″ W) and Carmona (37° 30′ 29″ N, 5° 34′ 42″ W). At Carmona, two different watering regimes were imposed: mild stress (MS) and irrigated (I), while rainfed conditions (SS) were maintained at Ecija. Irrigation and agricultural practices followed the local standard practices on rainfed (Ecija) and irrigated (Carmona) farms. The first irrigation at Carmona took place at day of year (DOY) 72 for both MS and I blocks (28 mm). A second irrigation at Carmona was made the day before sampling (DOY 119), but only for the irrigated (I) block (28 mm). By the sampling date, the accumulated precipitation was 317.4 mm for Ecija and 571.2 mm for Carmona. The meteorological conditions were very similar at the two locations, which enabled us to compare results between them (Fig. S1).

Physiological analyses and RNA-Seq sampling of the flag leaf (three biological replicates per plot) were carried out at grain filling (15 dpa, Zadoks stage Z77; Zadoks et al. 1974), simultaneously with high-resolution RGB, hyperspectral and thermal remote sensing imagery using an airborne platform (Fig. 1).

Physiological water status assessment (Fig. 1a, b) and nitrogen status sampling of the flag leaf were carried out in the field to characterize the three watering regimes. Leaf water potential (LWP; MPa) was measured using a pressure chamber (Model 600, PMS Instrument Company, Albany, USA) on three sunlit flag leaves per plot. The assimilation rate and stomatal conductance were measured with a portable photosynthesis measurement system (LCpro-SD, ADC Bioscientific Ltd., Herts, UK). Leaf reflectance was measured with a Polypen RP400 (Photon Systems Instruments, Drasov, Czech Republic) on ten sunlit leaves per plot. The leaf optical reflectance data were used to calculate the Photochemical Reflectance Index (PRI) and the Carotenoid Index (CAR) (Gamon et al. 1992; Zarco-Tejada et al. 2013a). Steady-state leaf chlorophyll fluorescence yield (Leaf F) was also monitored in ten sunlit leaves using a FluorPen FP1000 (Photon Systems Instruments, Drasov, Czech Republic).

The field plots represented three stress conditions [irrigated, and two drought stress levels (mild and severe stress)], as confirmed and quantified by physiological measurements and remote sensing analyses from both farm sites (Fig. 1a, b). Remote sensing indicators of water and nitrogen status in Mediterranean conditions were computed following Gonzalez-Dugo et al. 2015 (Crop Water Stress Index (CWSI)), Zarco-Tejada et al. 2013b (SIF via the FLD2 method), and Herrmann et al. 2010 (TCARI_1510_).

### RNA extraction, sequencing, and DGE analysis

Three flag leaf biological replicates were taken from the central part of the central row in each cv. “Chinese Spring” plot under severe stress (SS), mild stress (MS), and irrigation (I). Flag leaf tissue (Zadoks stage Z77) was frozen in dry ice in the field and kept at − 80 °C until RNA extraction using the RNA-easy Plant Mini Kit from Qiagen, following the manufacturer’s instructions. RNA integrity was checked in an Agilent 2100 Bioanalyzer and all samples had a RNA Integrity Number (RIN) greater than 7, except the third replicate of the irrigated plot (sample 9). Several RNA extractions were carried out for this sample, but the RIN remained at an unacceptably low level (4.5). Nevertheless, the sample was sequenced, as there was no possible replacement.

PolyA directional libraries were constructed using a NEBNext® Ultra™ Directional RNA Library Prep Kit for Illumina, following the manufacturer’s recommendations, with the following modifications: fragmentation size was reduced to 12 s for samples IAS_CS1 to IAS_CS8 and to 8 s for sample IAS_CS9, to obtain a sufficient fragment size for the paired-end sequencing. The libraries were constructed and sequenced by Fundación Parque Científico de Madrid, in an Illumina NextSeq 500 run in 2 × 75 paired-end format (v2 reagents), according to the manufacturer’s instructions.

RNA-Seq raw paired-end reads in stranded mode were submitted to the NCBI Sequence Read Archive platform with the study accession code SRP119300 and BioProject PRJNA412622. This platform contains nine libraries corresponding to the three levels of drought stress analyzed (i) SRS2563966-IAS_CS1to3 (severe stress), (ii) SRS2563967-IAS_CS4to6 (mild stress), and (iii) SRS2563968-IAS_CS7to9 (irrigated).

Quality assessment was carried out using FastQC software (version 0.11.3) to evaluate read quality. A particularly high rRNA contamination was identified in library SRX3240768-IAS_CS9 (~ 40% % ribosomal content), which agrees with the GC deviation and increased duplication levels found in library SRX3240768-IAS_CS9 quality assessment. From then on, library SRX3240768-IAS_CS9 was discarded from further analyses.

Differential gene expression analyses of the RNA-Seq data were carried out using RefSeqv1 gene models (IWGSC 2018), through two bioinformatic pipelines. The first consisted of Kallisto (version 0.43.0) and the R library sleuth (version 0.28.1), whereas the second pipeline was based on STAR and the R library DESeq2 (version 1.14.1). The gene set surpassing the *p*-adjusted *Q* value threshold 0.05 for any of the pipelines at any stress condition comprised 3687 genes and was used for all subsequent analyses. The number of genes DE in both pipelines applying the thresholds |lg_2_FC, β| > 1.0 and *p*-adjusted *Q* value < 0.05 was 979. Principal component analysis (PCA) was carried out using the library DESeq2 in R, showing a high inter-replicate variability (Fig. S2a) when compared with a controlled environment experiment dataset [NCBI SRA database: SRP045409 (Liu et al. 2015)] that was re-analyzed by applying our data filtering and thresholds (Fig. S2b).

The first PCA component accounted for 60.4% of the variance and correctly separated the three field drought stress levels (Fig. S2a). The integrated PCA of our samples and PEG-stressed samples (Fig. S2b) showed high separation for component PC1 due to the different nature of the two experiments (field stress vs. PEG drought shock in controlled conditions, plus variety and tissue age differences). The second component PC2 (explaining 19.5% of the variance, Fig. S2b) separated the controls and the two drought stress levels for both experiments, indicating common responses to drought stress.

To obtain a physical visual overview of the expressed and DE HC genes, in relation to the total genes in the wheat chromosomes, their values were calculated for each 10-Mb window (Fig. S3). The DE genes were those reported by Kallisto/sleuth or STAR/DESeq2, i.e., with |lg_2_FC| > 1.0 and *p*-adjusted < 0.05, or |β| > 1.0 and *Q* value < 0.05, respectively. The expressed genes considered are those with a TPM value above 0.5 in any sample of the control, or mild or severe stress levels. The total genes are the number of RefSeqv1 HC genes located in each 10-Mb window.

We also carried out a de novo DGE analysis of the publicly available wheat PEG shock RNA-Seq dataset [NCBI SRA database: SRP04549, (Liu et al. 2015)] using RefSeqv1 gene models with our bioinformatic pipelines and thresholds, revealing common and specific genes (Fig. 2).

The carotenoid biosynthesis pathway genes (Fig. S7a) were obtained using BlastKOALA (http://www.kegg.jp/blastkoala/) with different sets of genes. Gene enrichment analyses (Table S2, S3) were carried out using a one-sided Fisher’s exact test.

### miRNA analysis

RNA sequencing data from irrigated, mild, and severe drought stress samples were aligned on IWGSC RefSeq1 chromosome sequences with Tophat software (version 2.1.1). Aligned reads for each different condition were then assembled with StringTie (version 1.3.3b), after which the transcripts obtained from these assemblies were subjected to miRNA identification. The comparison of the transcripts produced by each of the three conditions against a set of previously identified miRNA sequences that were retrieved from miRBase (version 21, June 2014) (Kozomara and Griffiths-Jones 2011) was performed using a two-step homology-based in silico method, as previously described (Akpinar et al. 2015; Alptekin and Budak 2017). Potential miRNAs with at most one base mismatch different to known mature miRNAs were identified using SUmirFind in-house script, available at GitHub (https://github.com/hikmetbudak/miRNA-annotation).

Transcripts that were the candidate precursors for these miRNAs were then extracted, subjected to fold prediction by the UNAFold v3.8 algorithm (Markham and Zuker 2005) and checked for pre-miRNA characteristics using our second in-house SUmirFold script, also available at GitHub (https://github.com/hikmetbudak/miRNA-annotation).

Finally, precursor sequences that satisfied the previous criteria were subjected to additional evaluation: (i) no mismatches were allowed at Dicer cut sites, (ii) no multi-branched loops were allowed in the hairpin containing the mature miRNA sequence, (iii) mature miRNA sequence was required to be located at the head portion of the hairpin, (iv) no more than 4 and six mismatches were allowed in miRNA and miRNA*, respectively (Akpinar et al. 2015; Alptekin and Budak 2017; Lucas and Budak 2012). These analyses revealed 313, 375, and 318 unique mature miRNA sequences in samples of irrigated, mild stressed, and severe stressed plants, which spanned 37, 35, and 39 miRNA families, respectively (Fig. S6). In both drought stress-treated samples, miR5049, miR1130, miR1436, and miR1122 families contained the four highest numbers of identified miRNAs, where the members of these four families constituted 54% and 49% of all identified miRNAs from the samples collected from mild and severe drought stress, respectively. Almost half of the identified miRNAs (49.2%) from the irrigated control sample were included in five miRNA families: miR1127 along with the four families containing the highest miRNA numbers in drought stress conditions, which indicates a similarity between the most abundant miRNA families in the control and stress-treated samples (Fig. S6). The comparison of miRNAs between the three samples (Table S5) revealed the miR1118, miR1136, and miR9668 families as being expressed in both stress levels, but not in control conditions. By contrast, the miR1139, miR9666, and miR9776 families were expressed only in control conditions. In addition, whereas only the miR9654 family was specific to mild stress, five miRNA families (miR1125, miR397, miR5067, miR9772, and miR9781) were specific to severe stress. Two miRNA families, miR437 and miR5200, were identified from both irrigated control and severe stress levels, but not from mild stress-treated samples, which might be an indicator that some cellular mechanisms are being turned off when the plant first encounters drought, then turned on again while the level of stress increases. Moreover, despite being identified in different copy numbers, 29 miRNA families were expressed in all three conditions. The expression of miR1122, miR1130, miR1137, and miR1436 increased greatly in both stress levels when compared to the control, and miR1127, miR398, miR5175, and miR5181 were highly downregulated in conditions of stress.

To find the molecular mechanisms that these miRNAs regulate, target gene analysis was performed using the IWGSC RefSeqv1.0 gene models (Table S6). The prediction of potential target transcripts of the mature miRNAs identified was conducted using the psRNATarget online web-tool (http://plantgrn.noble.org/psRNATarget/) (Dai and Zhao 2011). These target transcripts were compared with a set of known *Viridiplantae* protein sequences using BLASTx and functional annotations were performed with Blast2GO software. The results revealed 669 miRNA-target pairs that contained 162 unique CDS sequences are targeted by 30 unique mature miRNAs under control conditions. MiRNAs identified from mild drought stress were potentially involved in 670 miRNA-target interactions, and 153 unique coding sequences were targeted by 25 unique miRNA sequences in these interactions. Finally, the prediction of miRNA targets in severe drought stress indicated the presence of 687 miRNA-target pairs containing 206 unique coding sequences and 31 unique mature miRNAs. The GO enrichment from the terms described in Ramírez-González et al. (2018) for the miRNA targets was calculated using GoSeq (Young et al. 2010) (Table S7).

### Analysis of co-expressed genes

We extracted the set of genes surpassing the p-adjusted *Q* value threshold 0.05 for any of the pipelines under any watering regime (3687 genes) and applied self-organizing maps (SOM) to classify them into groups or modules with similar expression (Törönen et al. 1999). For each gene, we had eight TPM values, three for severe stress, three for mild stress, and two for irrigated control; these values were reduced to only three by calculating the average TPMs in each stress level. To apply the SOM analysis, the dimensions of the grid were specified using the variance values given by a PCA; the resulting ratio, 1.64, suggested a size of 5 × 8. Figure S7A shows the resulting SOM analysis using the R package kohonen version 3.0.4, including the cluster dendrogram of the resulting 40 classification units. To select the final number of modules, we decided to trace a line through the dendrogram so the number of units in each branch crossed by the line was balanced (see Fig. S7a). Therefore, the whole set of genes was divided into five clusters (Fig. 3 left).

We then performed GO term enrichment analysis of the genes belonging to each module. The abundance of GO terms in the automatic annotation revealed many enriched terms (*p* value < 0.05 using the R library GoSeq v1.26.0), which were represented through a Tree Map using the REVIGO web application (Supek et al. 2011). To reduce the complexity of these Tree Maps, a GO Slim term enrichment analysis was carried out for each SOM module (Modules 1 to 5, see Fig. 3 left) and for all the DE genes under severe stress (reported by both Kallisto/sleuth and STAR/DESeq2, Fig. S8). The original GO annotation (IWGSC 2018) was processed to narrow it down to GO Slim terms only; then, in a first step, the GO Slim terms associated with stresses were added (as described in Ramírez-González et al. 2018). Finally, as a second step, in accord with the drought-oriented nature of this study and to obtain more specific data, the GO term associated to response to water (GO: 0009415) was added. The term enrichment was performed with Cytoscape version 3.5.1 (visualization) and its BiNGO plugin version 3.0.3 (analysis).

To study the behavior of the homoeolog genes in the context of the five modules created, i.e., to find out whether all the three homoeologs in ABD behaved similarly and fell in the same module, we retrieved all of the pure triads (those with homoeologs in the A, B, and D subgenomes in the form 1:1:1) containing at least one gene in the original set of 3687. The result was a set of 1470 triplets with 4410 genes. From them, the genes not belonging to the original set had to be fit manually into the most suitable module. However, the behavior of some of them was ambiguous and they did not clearly fit into any particular module; in these cases, a probabilistic neural network (PNN; R library version 1.0.1) was used to assign a module to each of them; in general, the PNN provided less significant results than the manual classification because it does not use specific knowledge from the problem being resolved. Once every gene of every triplet had been assigned a SOM module, the similarity of behavior could be obtained. Figure S7b shows that 57.7% of the triplets had three homoeologs belonging to the same module; the three homoeologs belonged to three different modules in only 4.7% of cases; for the other triplets, only one of the homoeologs behaved differently.

The weighted gene correlation network analysis (WGCNA) was carried out using CEMiTool version 1.4.0 (Russo et al. 2018) and WGCNA version 1.63 (Langfelder and Horvath 2008) applying variance stabilizing transformation (VST) to 61 samples (listed in Table S9) whose TPM values were obtained through Kallisto. The WGCNA obtained 30,180 genes with *p* < 0.1 distributed into 19 modules. These modules and their genes (Supplementary Table file SM1) were used as input for BiNGO obtain GO terms with enrichment at *p* < 0.05 using the biological process ontology. The annotation file used with BiNGO contained the HC and LC genes and their associated GO Slim terms enhanced with stress and water stress GO terms. The most relevant overrepresented GO Slim terms (*p* < 0.05 using Benjamini and Horchberg FDR) from both the SOM and WGCNA analyses are shown in Fig. 4.

### Clusters of drought-responsive genes

Using the RefSeqv1 gene models, all gene clusters made up of, at least, three HC genes with the same functional annotation within a 1-Mb window in each chromosome were identified and filtered according to the following criteria, adapted from the ARID [genomic regions hosting compact clusters of genes which are anhydrobiosis-related and accumulate transcripts upon desiccation] and cluster of desiccation-associated genes [CoDAG, (Costa et al. 2017)] gene cluster definitions:

(i) the set of genes contains homoeologs in the three subgenomes
(ii) their localization in the genome is not necessarily related to that of the potential ancestor of the expanded set of genes; and
(iii) at least one gene from the cluster is differentially expressed in field drought stress conditions (*p* < 0.05), and more than 50% of the genes in the cluster are DE in any of the available drought experiments (either field drought or under PEG treatment (Liu et al. 2015)) at (p < 0.05).

A total of 132 gene clusters contained field drought-responsive genes (at least one), involving a total of 2493 genes. From them, 51 clusters (including 915 genes) were considered CoDReGs, as they fulfilled the above requirements. From the 3058 (2661 HC) differentially expressed genes in this work (either in mild or severe stress using any pipeline), 141 of them are located in CoDReGs. Of these, 70.7% are located in R1/R3 regions, 29.3% in the R2a/R2b regions and none in the centromere. This is significant (*χ*^2^, *p* < 0.05 in a simple Fischer test), as it represents a ratio of 2.4× in R1/R3 vs R2a/R2b, whereas the proportion of HC genes in the genome is 1.21×. The CoDReG distribution in the five SOM clusters is shown in Table S10. Three CoDReGs present a complex structure, with two blocks of genes in one chromosome: CoDReG 4_456_01 contains two blocks in 4A; CoDReGs 3_712_01 and 6_005_01 contain two blocks in the D chromosome.

The whole-genome comparison between rice (42,132 genes, IRGSP 2005), barley (39,734 genes, Mascher et al. 2017), *Brachypodium* (31,029 genes, The International Brachypodium Initiative 2010), sorghum (36,388 genes, Paterson et al. 2009), and hexaploid wheat (IWGSC 2018) was performed individually for each of the wheat A, B, and D subgenomes (A 36,302 HC genes; B 36,738 HC genes; D 35,021 HC genes) using MCScanX with a threshold value = 10^−5^ and a size of synteny block = 10 genes (Wang et al. 2012). Retaining only the 1:1 relationships in each comparison, 9663, 13,551, 10,567, and 9399 orthologous relationships were identified between the A genome and the rice, barley, *Brachypodium*, and sorghum genomes, respectively. Similarly, we obtained 9314 (rice); 13,542 (barley); 10,420 (*Brachypodium*); and 9080 (sorghum) orthologs for the B genome and 9567 (rice); 14,049 (barley); 10,821 (*Brachypodium*); and 9407 (sorghum) orthologs for the D genome. The results for the dehydrin gene family are shown in Table S15. To investigate the roles of the 51 CoDReGs identified previously, we used the Q-TARO database (http://qtaro.abr.affrc.go.jp/), which revealed the chromosomal locations of many QTLs in the rice genome and their contributions to phenotypic variation (Yamamoto et al. 2012; Yonemaru et al. 2010). From the database, we extracted 111 QTLs containing the term “drought tolerance” distributed over the 12 rice chromosomes. Comparing the QTL locations and the 51 CoDReGs using the synteny relationships, we identified 28 of them as conserved drought tolerance QTLs. These results, including clusters, rice QTL locations, and synteny relationships, were represented in circle form using Circos software (Krzywinski et al. 2009) (Fig. S10). Gene enrichment on CoDReGs was tested using one-sided Fisher’s exact tests (Fisher 1922: see Table S3).

### Dehydrin gene family analysis

The 67 genes labeled as encoding dehydrins by the IWGSC automatic annotation process were retrieved from RefSeqv1 gene models (IWGSC 2018). We additionally performed a manual check and curation. As a double check, a BLAST search for the K-segment in the complete gene model retrieved 57 (50 HC + 7 LC) of the above genes. UniProt contained 58 (51 HC + 7 LC) wheat proteins from the above dehydrins and did not add any additional *DHN* genes.

We started the manual curation with an analysis of the three typical segments of wheat dehydrins (Malik et al. 2017). In proportion to their length, we allowed 4, 3, or no mismatches for the K-, Y-, and S-segments, respectively. This approach provided a more detailed view compared to a BLAST-based analysis alone, in order to represent the 67 putative *DHN* gene structures.

The gene triad in chromosome 1 and a doublet of the LC genes in chromosome 5 were discarded from further chromosomal analysis because they presented low BLAST quality scores, showed an absence of the three *DHN* segments (K, Y, and S), and did not have orthologs either in *Oryza sativa* or *Brachypodium distachyon*. In addition, we did not include the two LC genes located at the U chromosome because we could not infer their correct chromosomal location. However, the two remaining HC genes initially located at U chromosome could be positioned on chromosome 6B and chromosome 6D using the TGACv1 gene models (Clavijo et al. 2017). Figure S12 shows the gene structure of the remaining set of 60 genes used for further analysis. They had an average of 1.97, 0.87, and 0.72 K-, Y-, and S-segments per dehydrin, respectively, with a median of 2, 1, and 1 in chromosomes 5 and 6, and 3, 2, and 1 in chromosomes 3 and 4; dehydrins in chromosome 7 were missing the Y- and S-segments.

An update of dehydrin names was carried out as follows:

(i) The RefSeqv1 annotation provides the best BLAST hit against several species (not including wheat). Starting from such a hit, we obtained the associated entries in EMBL/EBI and the corresponding names of dehydrins, when available.
(ii) We paired the dehydrins being studied (using BLAST) with those dehydrins known in UniProt, thus obtaining their names, when available.
(iii) We compared both results, assigning the following matching names: *DHN33*, *DHN6*, *DHN2*, *DHN1*, *DHN11*, *DHN4*, *DHN9.6*, and *DHN14*.
(iv) *DHN3* and *DHN5* are similar to many genes on chromosome 6. To avoid creating new numbers for them, we assigned a sub-numbering: from *DHN3.1* to *DHN3.8* and from *DHN5.*1 to *DHN5.4*.
(v) We numbered the rest of dehydrins with new numbers starting from 35 (the highest number already found among dehydrins was 33, so we decided to start with a round number). The names assigned ranged from *DHN35* to *DHN38*. The genes whose homoeology was not clear were numbered with “.1” or “.2” as a suffix (e.g., *DHN38.1* and *DHN38.2*).
(vi) We adapted these names to comply with (McIntosh et al. 2013).

Each specific dehydrin gene name contains the name of its dehydrin group, followed by the genome it belongs to: e.g., *DHN4-A1*, *DHN4-B1*, and *DHN4-D1*. If several genes in the same group belong to the same genome, a new incremental number is used, e.g., *DHN3-B1* and *DHN3-B2*.

A cluster of dehydrins was found as a result of the CoDReGs analysis (CoDReG 6_581_01), and it was studied in depth. The location of wheat MQTLs on 6L chromosome arms was compared to the 6L DHN cluster position of CoDReG 6_581_01. Wheat MQTLs (Acuña-Galindo et al. 2015) were located by BLAST searches using their flanking and representative marker sequences against RefSeqv1 (IWGSC 2018). Marker sequences were located online using graingenes.org and t3sandbox.org. BLAST results were filtered by (i) > 97% identity match; (ii) for SSR markers, forward and reverse primers were required to have a match on the right chromosome, be separated by less than 600 bp and have different orientation (strand “1” or “− 1”) in the BLAST results.

To identify putative regulators of dehydrin expression, we used a genie3 (Huynh-Thu et al. 2010) network, which predicts the downstream targets of TFs. The network was generated using 850 RNA-Seq samples from diverse tissues, ages, cultivars, and stress conditions (see methods in Ramírez-González et al. (2018)). In order to exclude weak interactions, we considered the top one million edges for further analysis. TFs were annotated using the domain architecture inferred via a phylogenomic approach (see methods in Ramírez-González et al. (2018)). We were able to identify putative regulatory TFs for 52 dehydrins, which included 228 TFs. We identified significantly enriched TF families that target dehydrins compared to all other downstream genes using *χ*^2^ tests.

## Electronic supplementary material

ESM 1 (XLSX 448 kb)

ESM 2 (PDF 7.57 mb)
