# Supplementary material for: Hotspots in the genomic architecture of field drought responses in wheat as breeding targets
Source: Funct Integr Genomics. 2018 Nov 16;19(2):295–309. doi: 10.1007/s10142-018-0639-3 (PMC6394720; doi:10.1007/s10142-018-0639-3)
Supplement: Supplementary file 2 — (PDF 7.57 mb) [file 10142_2018_639_MOESM2_ESM.pdf]

Supplementary Materials for

# Hotspots in the genomic architecture of field drought responses in wheat as breeding targets

[1*]Sergio Gálvez, [2]Rosa Mérida-García, [2]Carlos Camino, [3]Philippa Borrill, [4,5]Michael Abrouk, [3]Ricardo H. Ramírez-González, [6]Sezgi Biyiklioglu, [7]Francisco Amil-Ruiz, [8]The IWGSC, [9]Gabriel Dorado, [6]Hikmet Budak, [2]Victoria Gonzalez-Dugo, [2*]Pablo J. Zarco-Tejada, [10]Rudi Appels, [3*]Cristobal Uauy, [2*]Pilar Hernandez

**This PDF file includes:**

Figs. S1 to S16
Tables S1 to S17

a

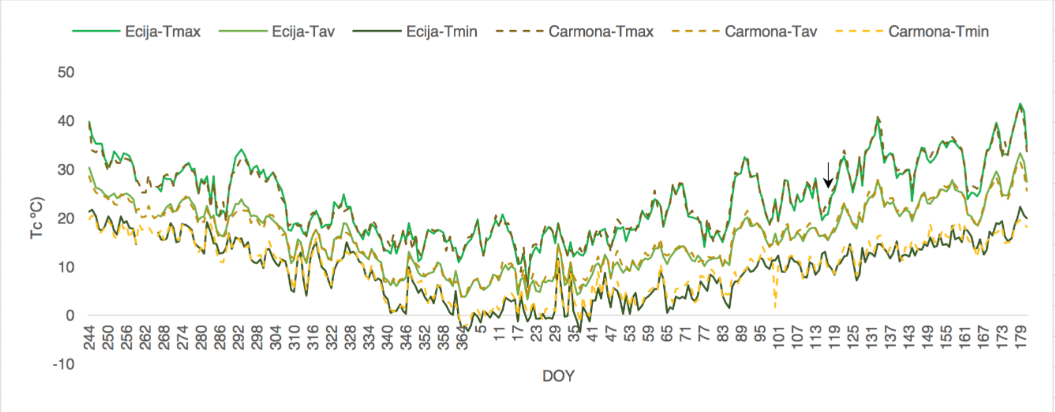


b

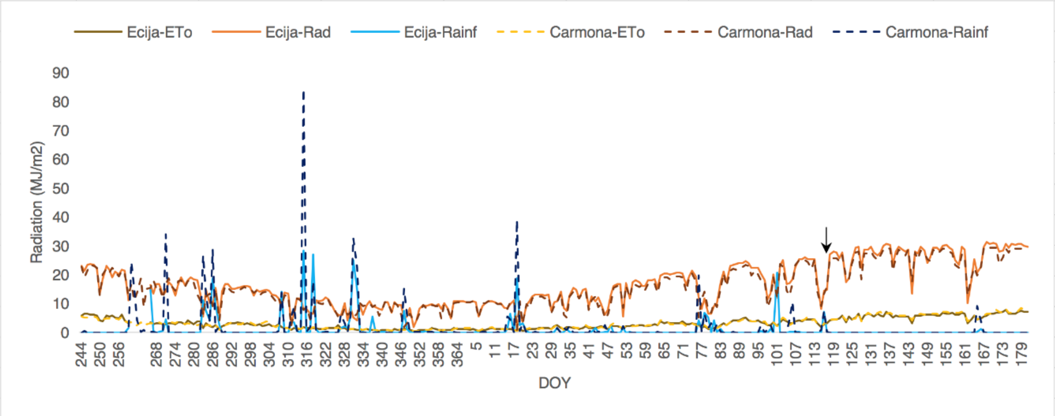


**Fig. S1. Weather characteristics of field sites used in this study.** (**a**) Maximum (Tmax), average (Tav), and minimum (Tmin) daily temperature (°C) during the growing season at both trial sites (Carmona and Ecija). (**b**) Evapotranspiration (ETo, mm), solar radiation (MJ $m^{-2}$), and rainfall (mm) for the same period. Arrows indicate the time of flight (on day of year (DOY) 120).

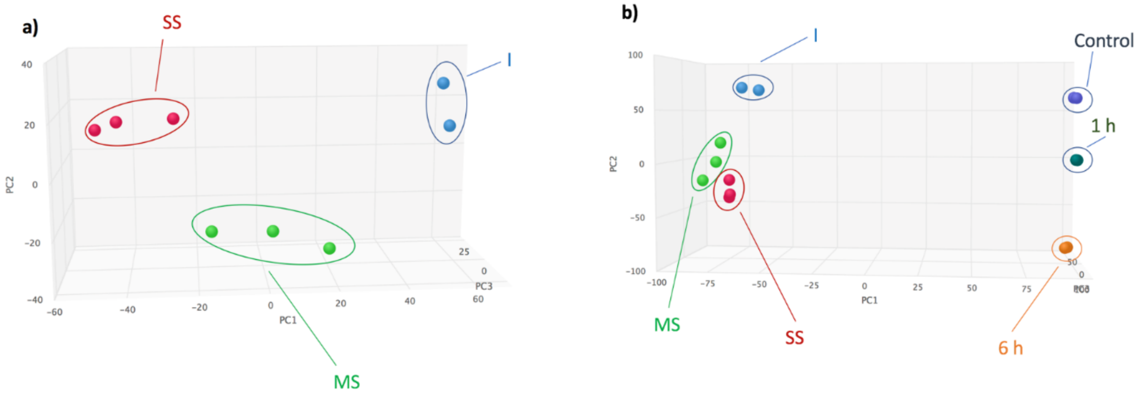


**Fig. S2. Principal Component Analysis (PCA) of RNA-Seq data. (a)** 3D PCA of RNA-Seq samples from this study. An interactive view can be accessed at <http://galactus.uma.es/galvez/triticum/ PCA3D_CS_best_pAdj.html> (**b**) Integrated analysis of samples from this study and the previously published work on PEG-induced drought in wheat seedlings (SRA SRP04540; Liu et al. 2017).SS: Severe stress; MS: mild stress; I: Irrigated; 1 h: one hour PEG stress; 6 h: 6 h PEG stress. An interactive view can be accessed at <http://galactus.uma.es/galvez/triticum/ PCA3D_CS_shock_best_pAdj.html>

**a**

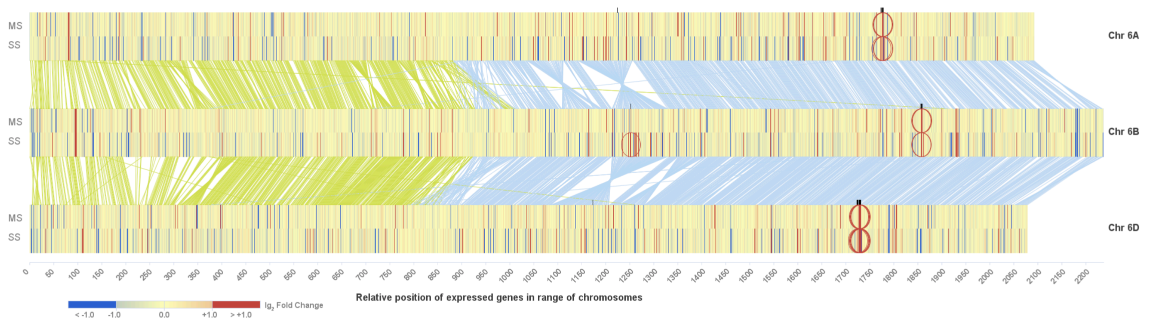


**b**

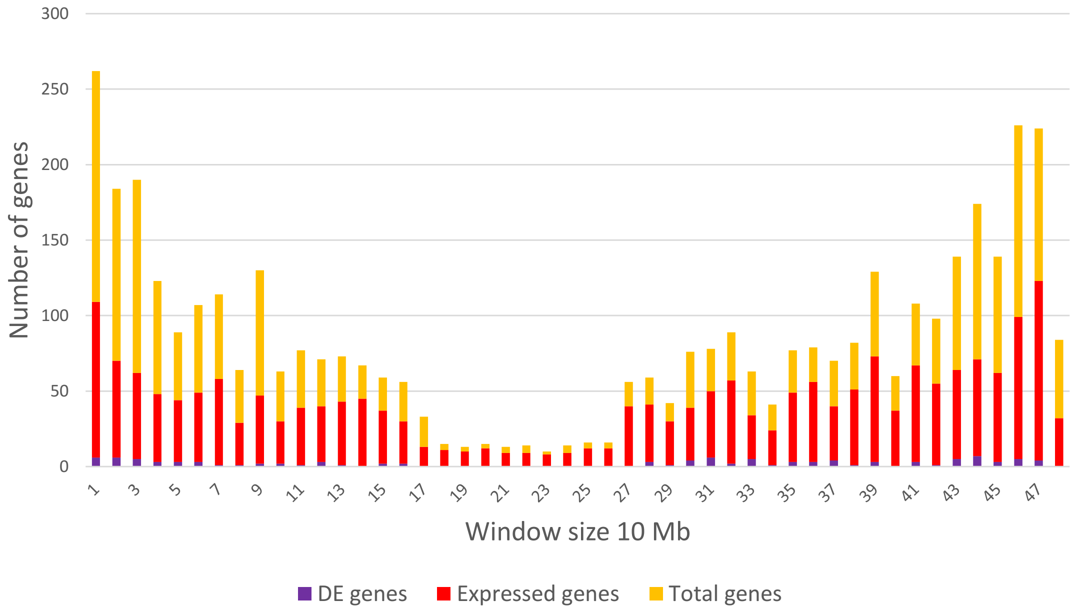


**Fig. S3. Physical distribution of DE genes in group 6 chromosomes.** (**a**) Gene expression map of differentially expressed genes under field drought conditions on group 6 chromosomes. Red circles indicate the physical position of *Dehydrin* genes. Green lines connect short arm homeologs, blue lines connect the long arm homeologs. MS: mild stress; SS: severe stress. (**b**) Physical position of DE HC on chromosome 6D. Expressed genes (red), DE genes (purple) and total genes (yellow) are shown per 10 Mb window.

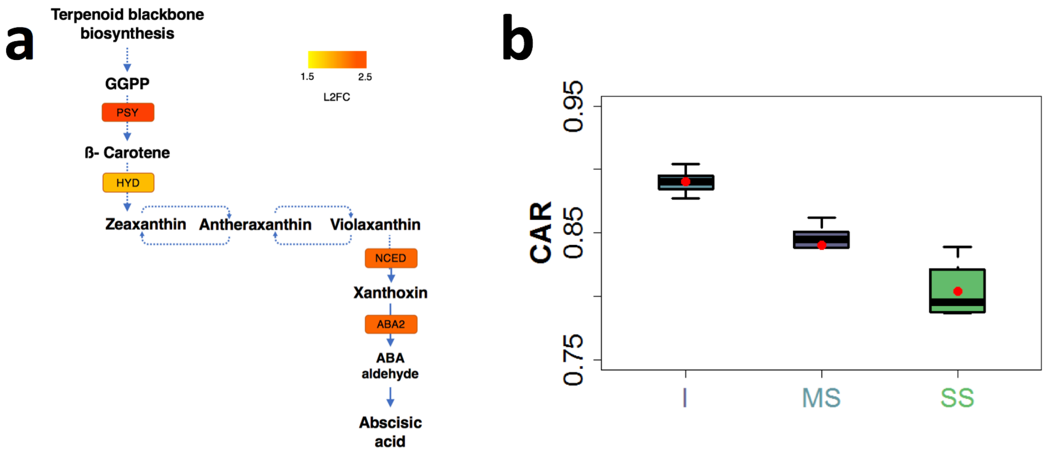


**Fig. S4. Transcriptional changes reflect remote sensing physiological data.** (**A**) Response of genes in the carotenoid biosynthesis pathway to drought stress. Overexpressed genes are shown in orange. (**B**) Chlorophyll Absorption Region (CAR) remote sensing trait corresponding to the three water status levels analyzed (I: irrigated; MS: mild stress; SS: severe stress). Boxplots include the median interquartile range and means (red dot).

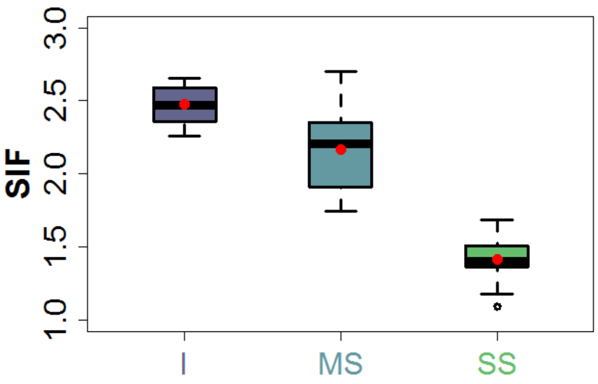


**Fig. S5. Solar-induced chlorophyll Fluorescence (SIF) remote sensing trait.** Boxplots include the median (bold horizontal line), 25-75 inter-quartiles range and means (red dots). Irrigated (I), mild stress (MS) and severe stress (SS).

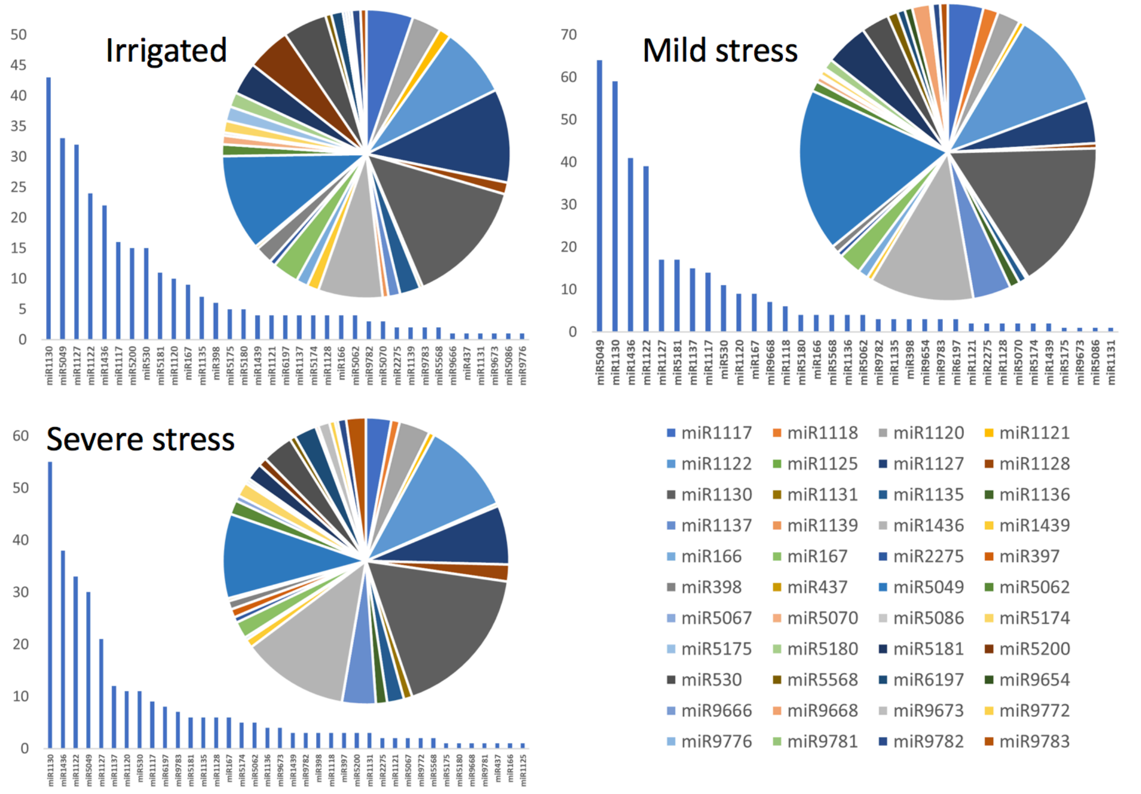


**Fig. S6. miRNA families and abundances identified from irrigated and drought stress samples.**

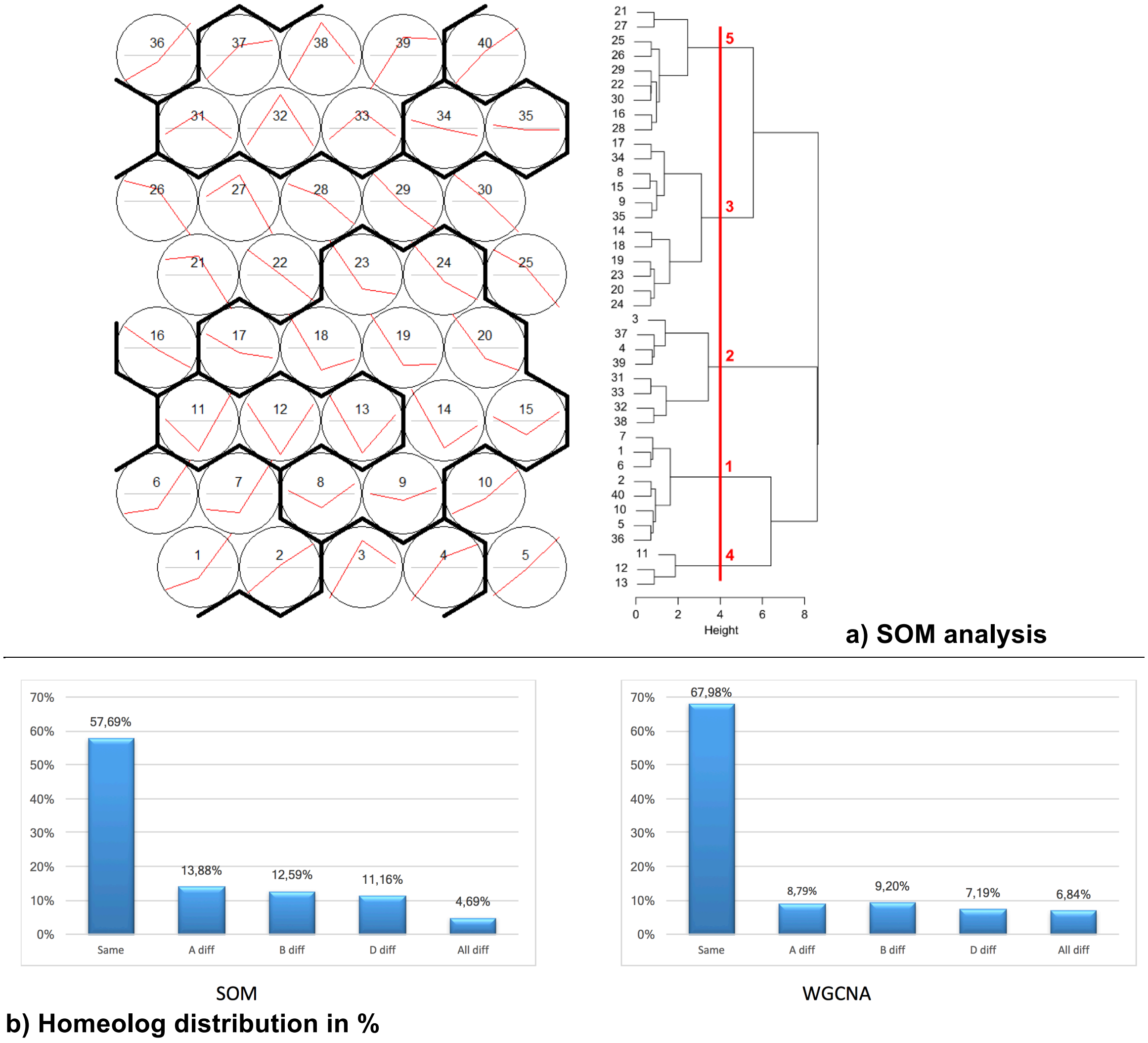


**Fig S7. Self-organizing map modules.** (**a**) SOM genes classified into 40 main patterns with a hierarchical organization which defines the five main SOM modules used in the analyses. (**b**) Distribution of homeologs in the five SOM modules (left) and in the 19 WGCNA modules (right). Left-hand bar shows the percentage of homeolog triads that are assigned to the same module; middle bars show the percentage of homeologs where one gene of the triad is assigned to a different module; right-hand bar show the percentage of homeolog triads where all genes are assigned to a different module.

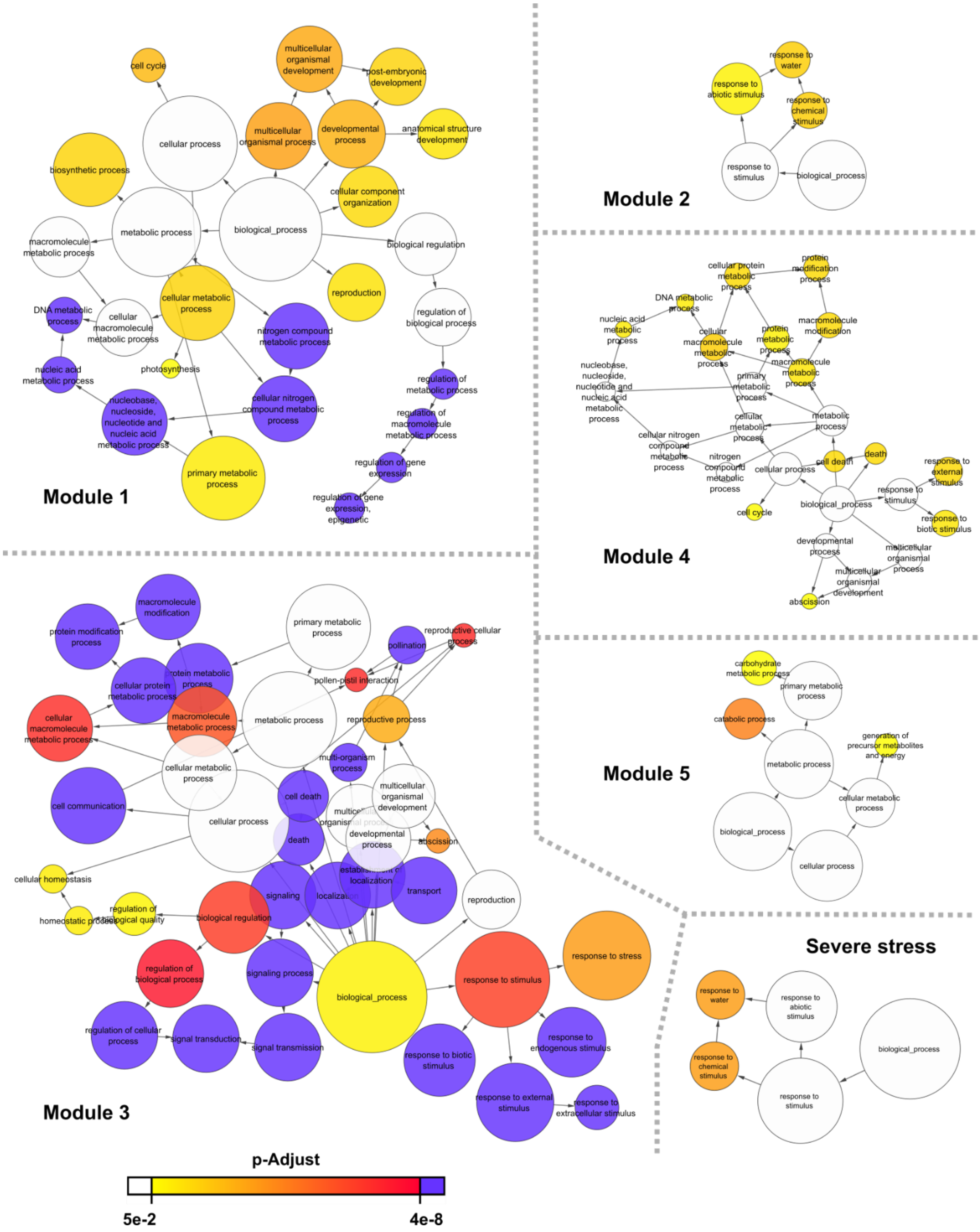


**Fig. S8. GO Slim terms enrichment analysis of the five SOM modules and for all DE genes under severe stress.** The size of a node is proportional to the number of genes annotated).

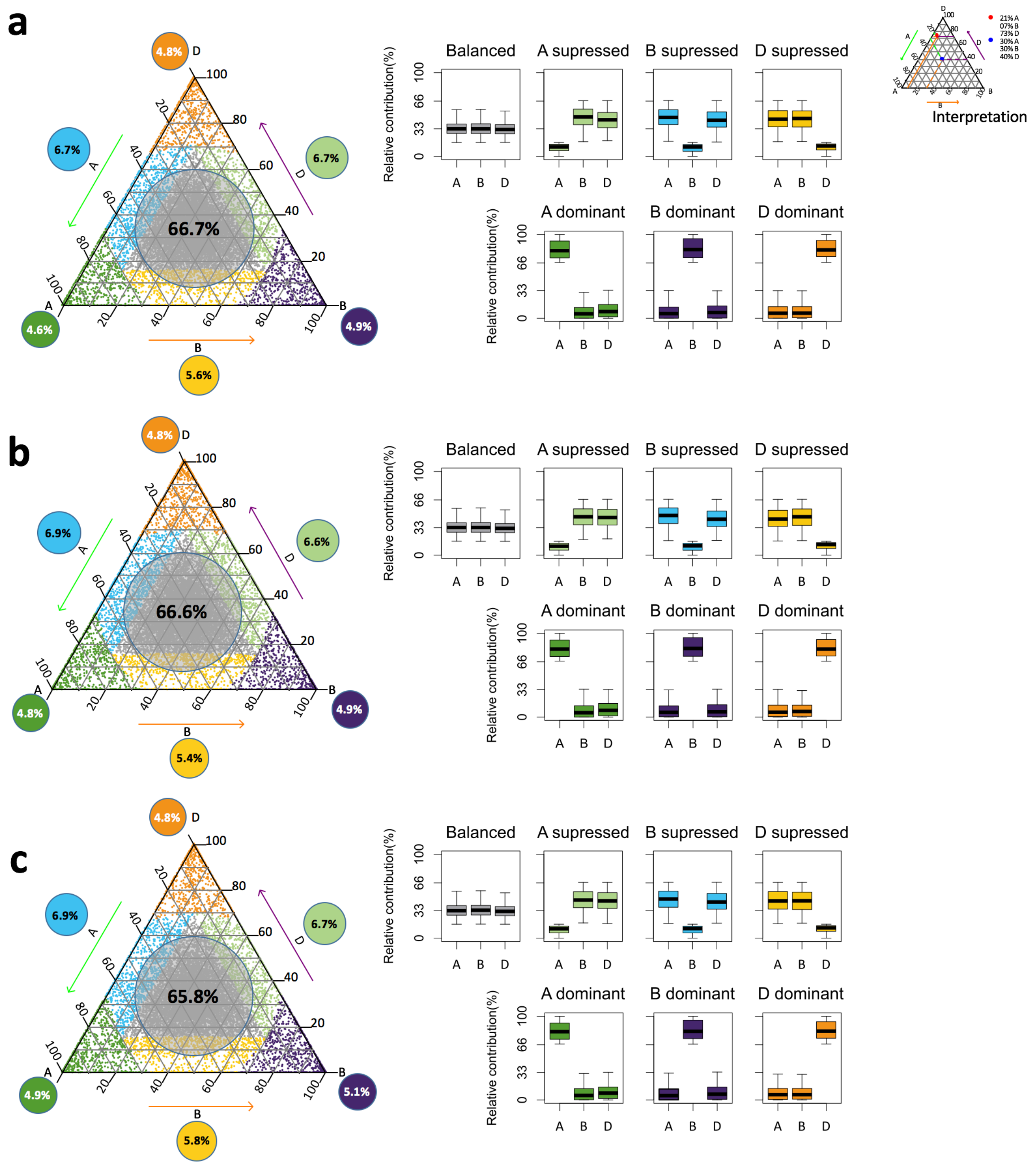


**Fig. S9. Relative homoeolog expression bias in genome triads (TPM>0.5). (a)** irrigated samples (13575 triads); (**b**) mild stressed samples (13904 triads), (**c**) severe stressed samples (13517 triads). Homoeolog triads are classified into 7 categories: (A, B, D)-dominant when the expression of a single homoeolog exceeds the 66%; (A, B, D)-suppressed when the expression of a single homoeolog is lower than 16%; and Balanced in any other case. Each triad is located in a spot corresponding to the percentage contribution of its constitutive genes, as shown in the Interpretation panel.

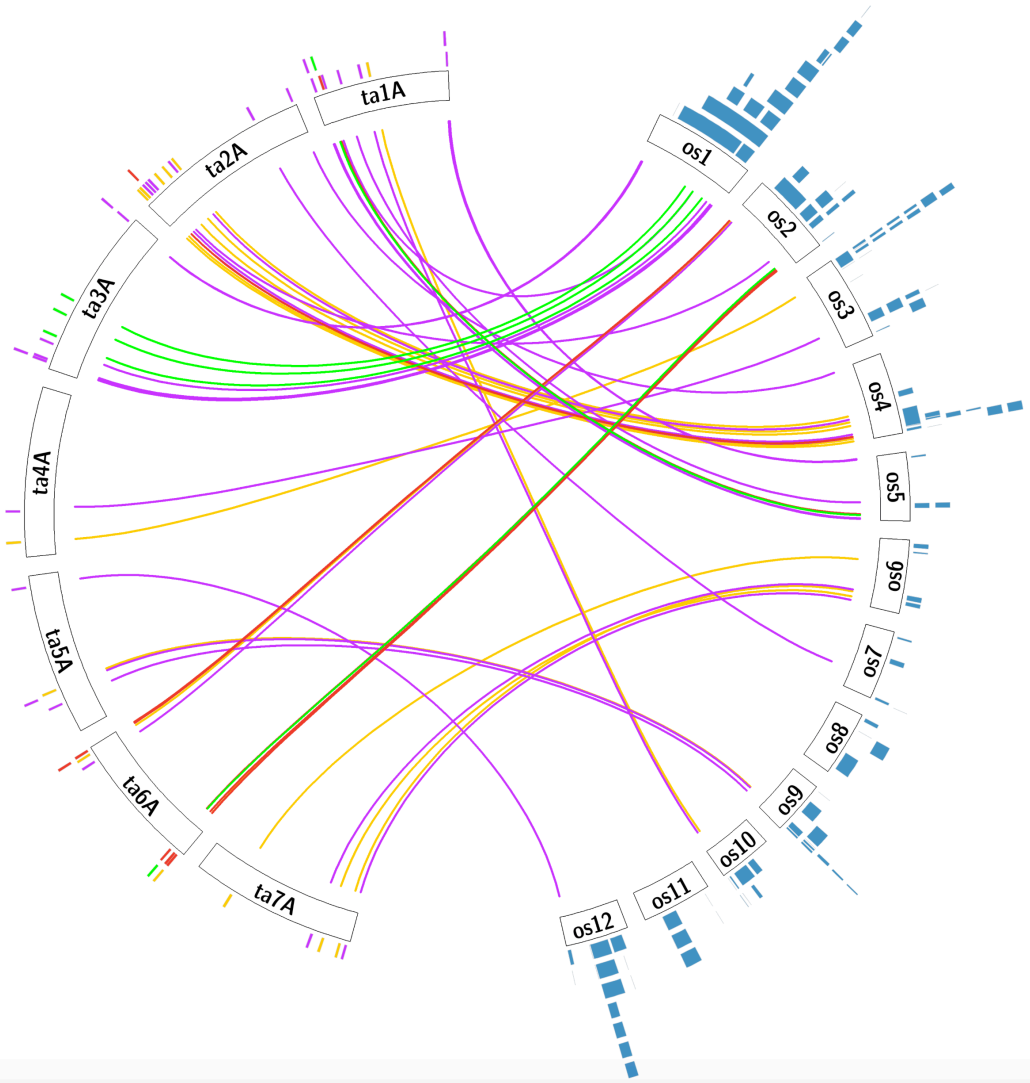


**Fig. S10. Circos plot representing the relationship between wheat CoDReGs (ta, left) and rice drought QTL intervals (os; right).** The seven chromosomes of the A genome are represented on the left-hand side and the twelve rice chromosomes are represented on the right-hand side. The CoDReGs location in the A subgenome is shown on the left of the wheat chromosomes. On the rice genome, the 111 QTL intervals related to drought-tolerance extracted from QTARO database (Yonemaru et al. 2010) are shown in blue. The clusters related to the SOM module 1 are marked in red; green for module 2; purple for module 3 and orange for module 5.

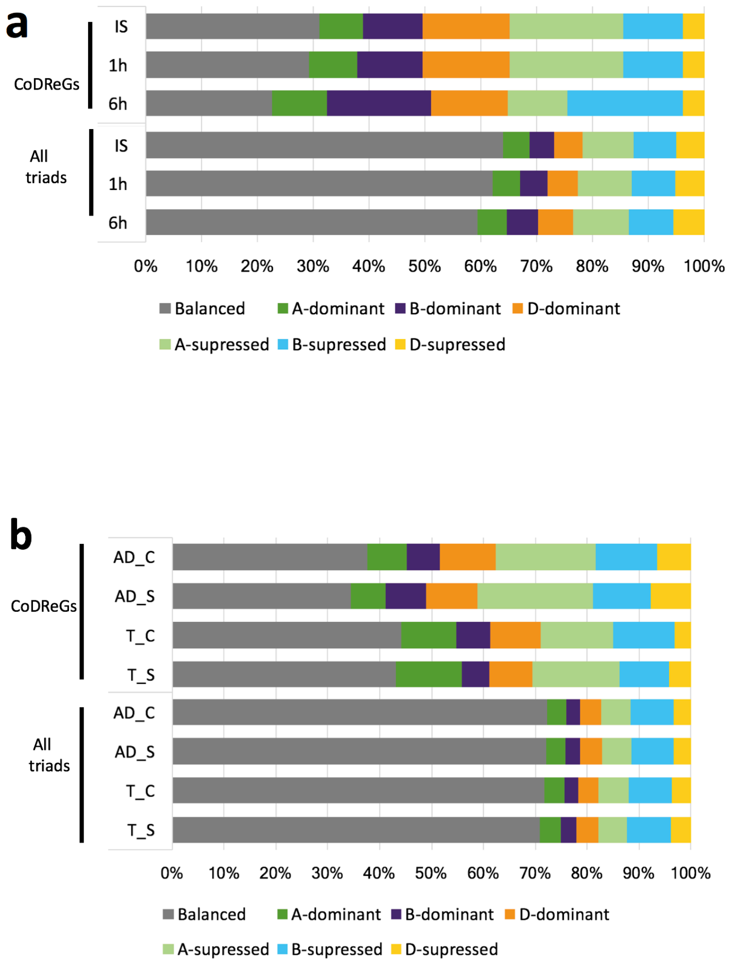


**Fig. S11. Homeolog gene expression bias** (corresponding to the seven classes categories described in Fig. S13) across the genome and CoDReGs, and the stress levels in (**a**) drought shock experiment in seedlings (Liu et al. 2017) and (**b**) rain shelter experiment in flag leaf (Ma et al. 2017).

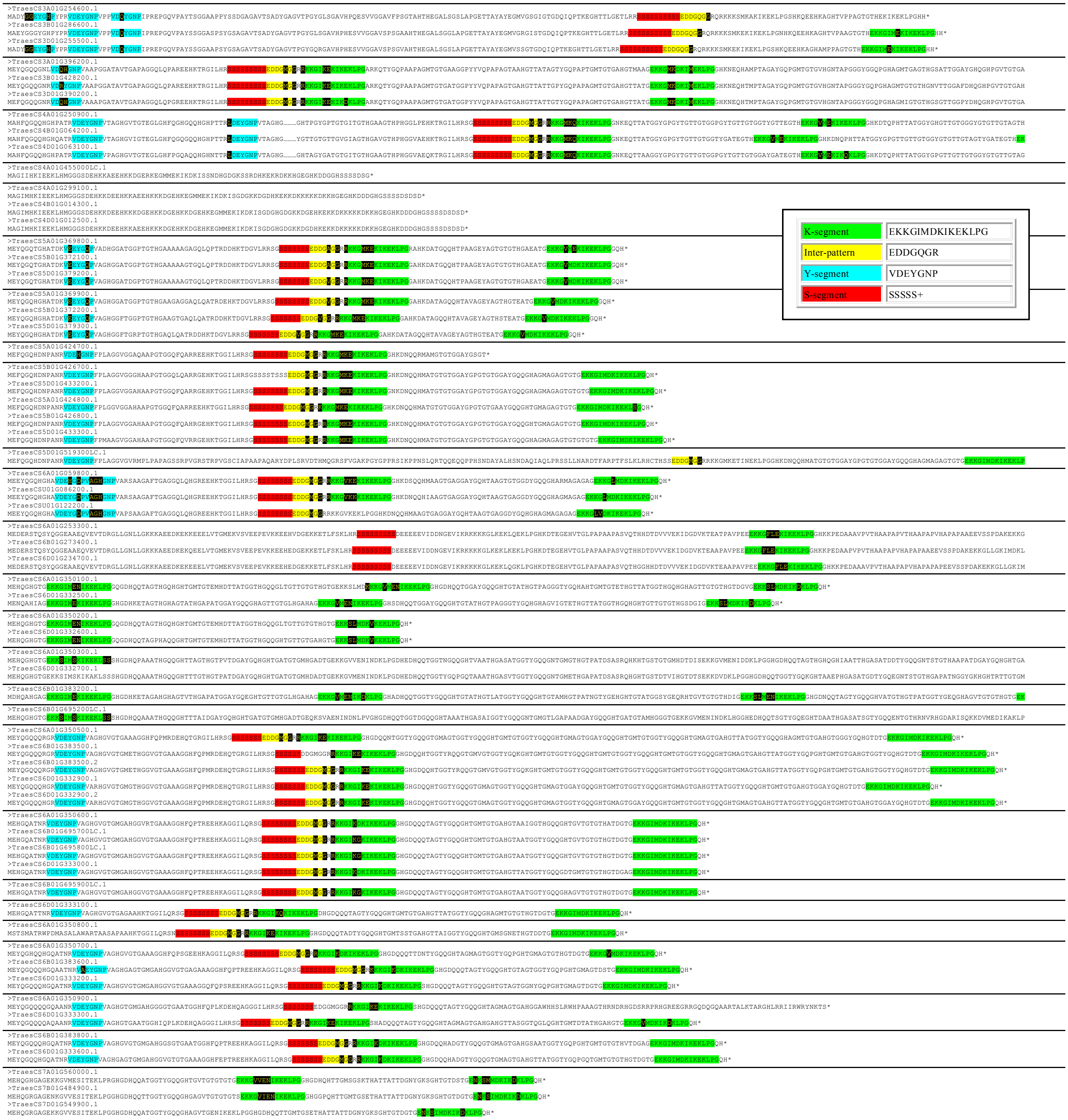


**Fig. S12. Structural analysis of wheat dehydrins**. The K-, Y- and S- segments contained in each dehydrin have been highlighted in green, blue and red, respectively. In addition, the inter-sequence pattern (EDDGQGR) has also been marked in yellow. Where necessary,

proteins have been truncated to show the initial amino acid sequence. The left side of the figure shows the chromosome assignment of each dehydrin.

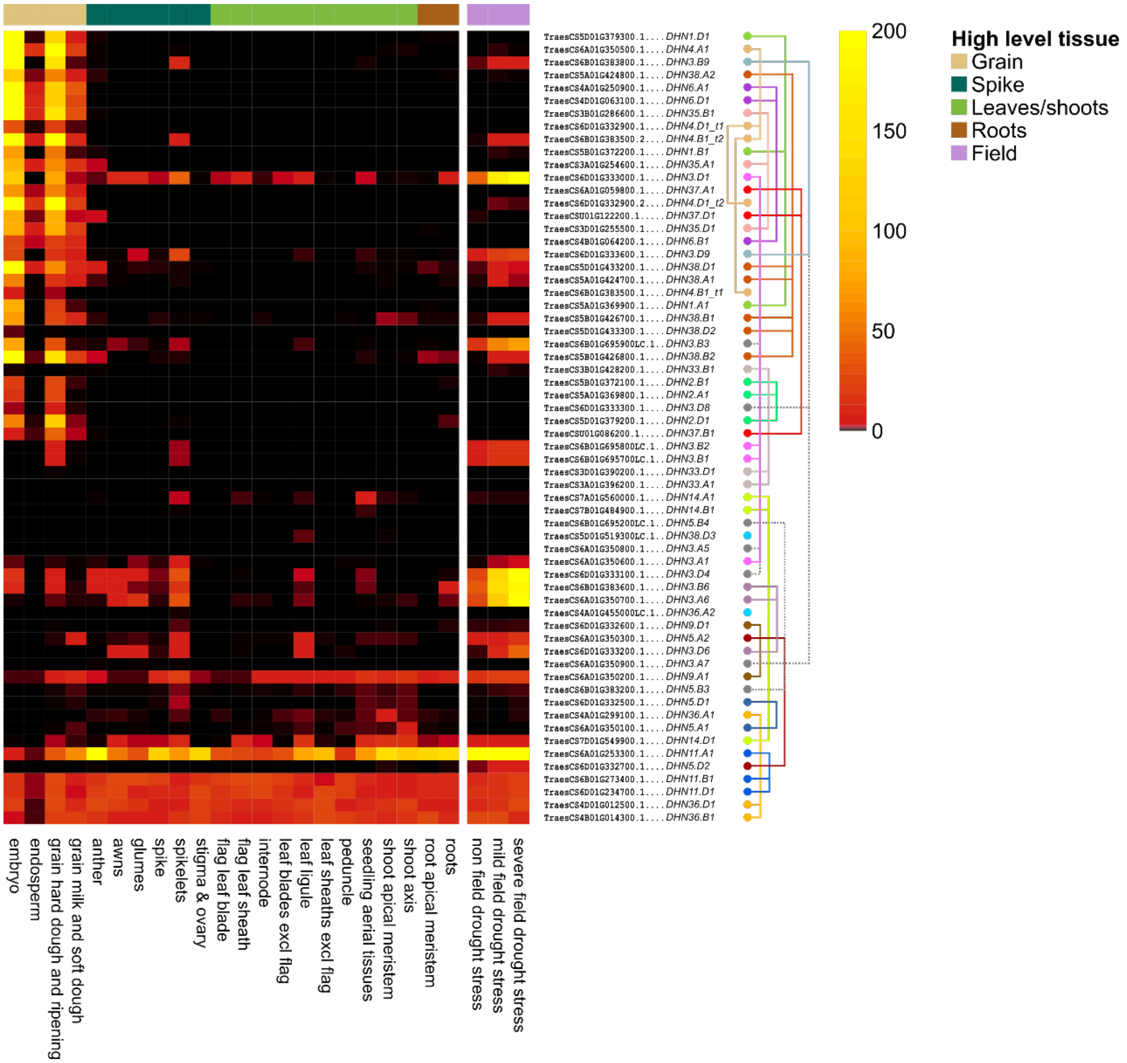


**Figure S13. Dehydrin genes clustering**. Figure is based on expression values of all available experiments at www.wheat-expression.com (left-clustering). The range of expression shown is 0 to 200 TPM; any TPM>200 has been set to 200. Columns indicate high level tissue grouping; the three non-clustered columns on the right represent the field samples. RefSeq1 gene IDs and their corresponding proposed gene names are shown. Colored lines connect homoeologs.

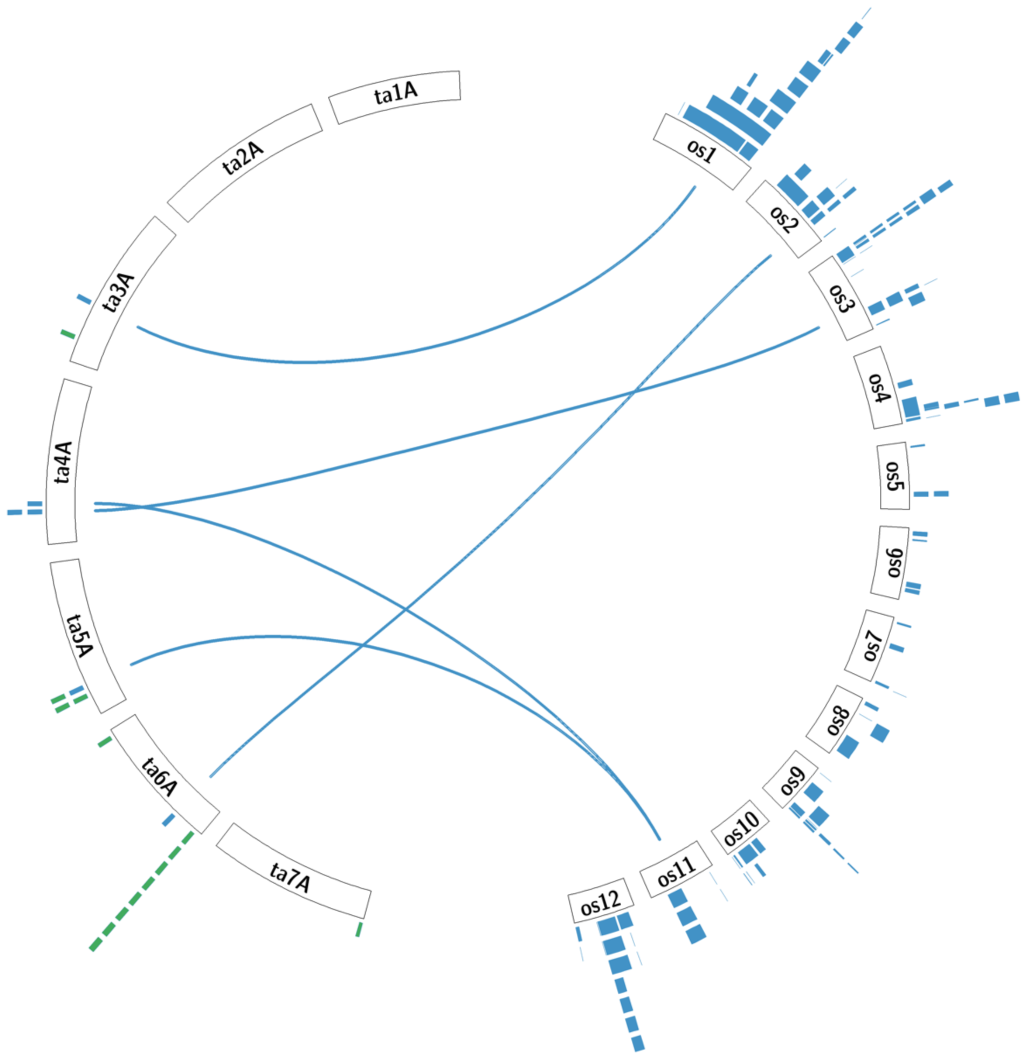


**Figure S14. Syntenic relationships between dehydrin genes of wheat (ta; left) and rice (os; right).** The seven chromosomes of the A genome are represented on the left-hand side and the twelve rice chromosomes are represented on the right-hand side. Ortholog (6) and non-ortholog (14) DHN genes located in wheat A subgenome are shown in blue and green, respectively. On the rice genome, the 111 QTL intervals related to drought-tolerance extracted from QTARO database (Yonemaru et al. 2010) are shown in blue. The blue lines between the two genomes represent the syntenic relationships of the DHNs.

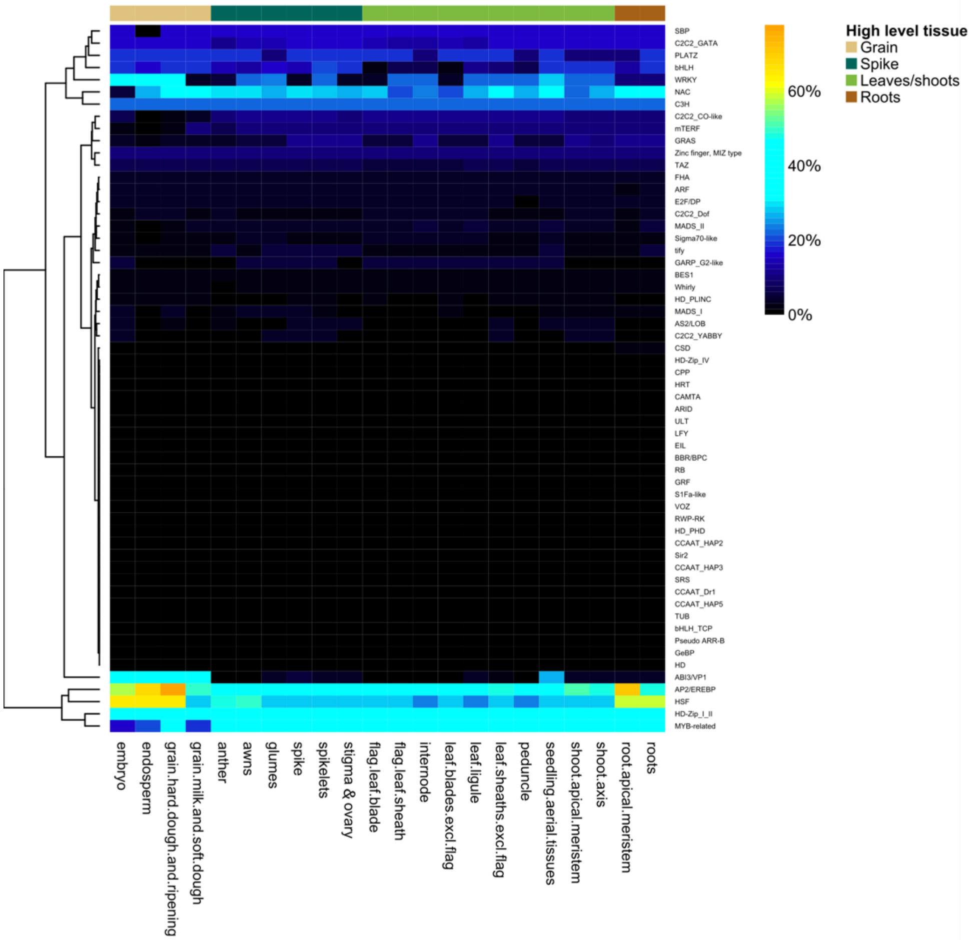


**Fig. S15. The regulation of dehydrins by transcription factor families.** The transcription factors which target dehydrins were predicted by genie3 analysis using the top 1 million edges (Ramírez-González et al. 2018). The percentage of high-confidence dehydrins regulated by each TF family are shown. Each row represents one TF family, with each column representing one tissue.

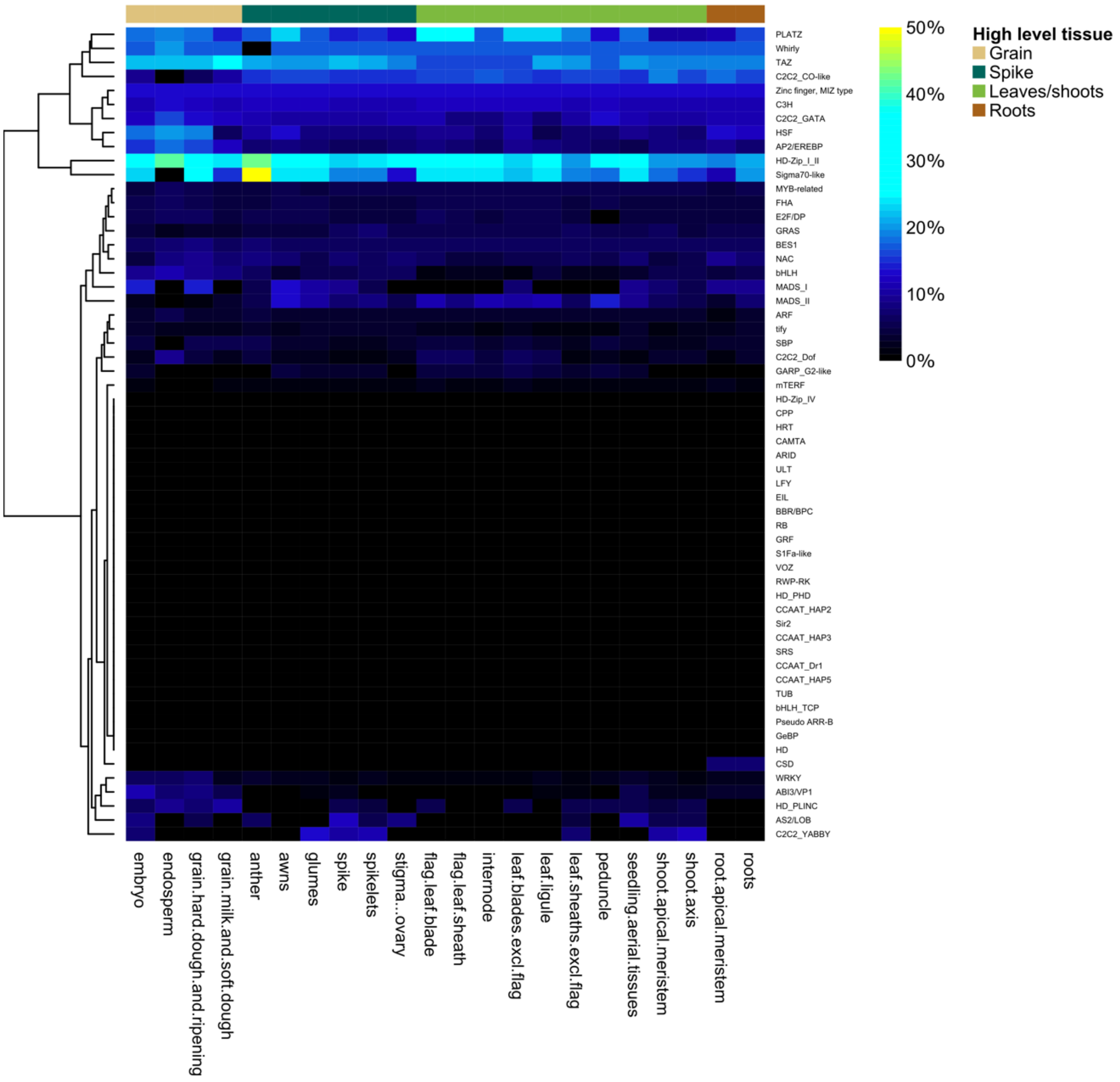


**Fig. S16. TFs predicted to regulate dehydrins.** The percentage of TFs which are predicted to regulate dehydrins by genie3 analysis using the top 1 million edges (Ramírez-González et al. 2018). Each row represents one TF family, with each column representing one tissue. Only TFs expressed ≥ 0.5 TPM were included in the analysis.

**Table S1.**

Analysis of variance results for remote sensing traits.

| | Mean ±Stdev | | | ANOVA | |
|---|---|---|---|---|---|
| | *I* | *MS* | *SS* | F value | p-value |
| **CWSI** | 0.13±0.040 | 0.51±0.074 | 0.64±0.060 | 369.5 | <2e-16 |
| **SIF** | 2.47±0.124 | 2.16±0.300 | 1.41±0.146 | 174.5 | <2e-16 |
| **$TCARI_{1510}$** | -0.25±0.025 | -0.15±0.014 | -0.10±0.004 | 86.49 | 6.52e-06 |
| **CAR** | 0.89±0.009 | 0.84±0.022 | 0.80±0.024 | 27.18 | 2.27e-05 |
| **PRI** | -0.61±0.040 | -0.53±0.040 | -0.51±0.030 | 11.71 | 0.00124 |

**Table S2.**
Differentially expressed (DE) and total gene distribution along chromosomal compartments (IWGSC 2018)[12].

| | DE genes (HC/LC) | % | Total genes (HC/LC) | % |
|---|---|---|---|---|
| **R1/R3** | 526 (471/55) | 53.7 (48.1/5.6) | 133,177 (57,116/76,061) | 49.4 (21.2/28.2) |
| **R2a/R2b** | 420 (380/40) | 42.9 (38.8/4.1) | 109,335 (46,582/62,753) | 40.6 (17.3/23.3) |
| **C** | 33 (28/5) | 3.4 (2.9/0.5) | 26,961 (7,092/19,869) | 10.0 (2.6/7.4) |
| **Total** | 979 (879/100) | 100 (89.8/10.2) | 269,473 (110,790/158,683) | 100 (41.1/58.9) |

**Table S3.**
Gene enrichment analysis in DE genes, ABD clusters, CoDReGs, dehydrin (DHN) and aquaporin (AQP) gene families.

| | DE genes | Genes in CoDReGs | DE genes in CoDReGs | DHNs | DE DHNs* | AQPs | DE AQPs* | Wheat genome |
|---|---|---|---|---|---|---|---|---|
| **Total Nº. of genes R1/R3** | 526 | 647 | 45 | 48 | 12 | 73 | 4 | 133,177 |
| **Total Nº. of genes R2a/R2b/C** | 453 | 268 | 8 | 12 | 1 | 89 | 4 | 136,296 |
| | P = 0.007 | P = 0 | P = 2e-6 | P = 0.001 | P = 0.007 | | | |

P: Significant exact Fisher test P values are indicated for significant tests. Gene decrease is shown with a red cell, gene enrichment is shown with a blue cell.
*By any bioinformatic pipeline.

**Table S4.**

DGE analysis significance parameters for differentially expressed Glutamine Synthetase genes, Photosystem I genes, the Photosystem II regulator PsbQ and UDP-glucose-6-dehydrogenase. Significant values (|lg$_2$FC, β|>1.0 and p-Adjust, Q-value<0.05) have been highlighted.

| | | Field | | | | | | | | PEG shock | | | |
|---|---|---|---|---|---|---|---|---|---|---|---|---|---|
| | | Mild stress | | | | Severe stress | | | | One hour | | Six hours | |
| | | Kallisto | | STAR | | Kallisto | | STAR | | STAR | | STAR | |
| Gene name | Description | Beta | Q-Val | L2FC | p-Adj | Beta | Q-Val | L2FC | p-Adj | L2FC | p-Adj | L2FC | p-Adj |
| TraesCS6D01G383600LC.2 | Glutamine synthetase GS1c: DQ124211 | | | -1.23 | 0.00 | | | -1.03 | 0.00 | -0.35 | 0.00 | -0.39 | 0.00 |
| TraesCS6D01G383600LC.1 | Glutamine synthetase GS1c: DQ124211 | -0.88 | 0.01 | -1.23 | 0.00 | -0.75 | 0.02 | -1.03 | 0.00 | -0.35 | 0.00 | -0.39 | 0.00 |
| TraesCS6B01G500000LC.1 | Oxygen-evolving enhancer protein 3-2, chloroplastic. Photosystem II regulator PsbQ | 1.06 | 0.22 | 1.32 | 0.11 | 1.61 | 0.00 | 1.99 | 0.00 | -0.11 | 0.90 | 2.01 | 0.00 |
| TraesCS3B01G667200LC.1 | Photosystem I iron-sulfur center | 0.76 | 0.79 | 0.55 | | 1.37 | 0.04 | 0.63 | | -1.06 | | 0.21 | 0.90 |
| TraesCS5A01G218200LC.1 | Photosystem I iron-sulfur center | -4.86 | 0.00 | | | 1.71 | 0.42 | | | -0.22 | | -0.15 | |
| TraesCS5A01G394900.1 | UDP-glucose 6-dehydrogenase: UDPg | -0.48 | 1.00 | -0.7 | 0.71 | -1.24 | 0.25 | -1.53 | 0.01 | 3.05 | 0.00 | 2.35 | 0.00 |
| TraesCS5B01G399800.1 | UDP-glucose 6-dehydrogenase: UDPg | -0.79 | 0.97 | -0.8 | 0.64 | -1.71 | 0.08 | -1.97 | 0.00 | 3.37 | 0.00 | 1.50 | 0.00 |
| TraesCS5D01G404300.1 | UDP-glucose 6-dehydrogenase: UDPg | -0.83 | 1.00 | -0.8 | 0.71 | -1.75 | 0.24 | -1.93 | 0.01 | 3.19 | 0.00 | 1.01 | 0.00 |

**Table S5.**
Comparative abundance of miRNA families between control, and stress-applied samples**.**
I: irrigated control; MS: mild stress; SS: severe stress.

| | I | MS | SS |
|---|---|---|---|
| miR1117 | 16 | 14 | 9 |
| miR1118 | 0 | 6 | 3 |
| miR1120 | 10 | 9 | 11 |
| miR1121 | 4 | 2 | 2 |
| miR1122 | 24 | 39 | 33 |
| miR1125 | 0 | 0 | 1 |
| miR1127 | 32 | 17 | 21 |
| miR1128 | 4 | 2 | 6 |
| miR1130 | 43 | 59 | 55 |
| miR1131 | 1 | 1 | 3 |
| miR1135 | 7 | 3 | 6 |
| miR1136 | 0 | 4 | 4 |
| miR1137 | 4 | 15 | 12 |
| miR1139 | 2 | 0 | 0 |
| miR1436 | 22 | 41 | 38 |
| miR1439 | 4 | 2 | 3 |
| miR166 | 4 | 4 | 1 |
| miR167 | 9 | 9 | 6 |
| miR2275 | 2 | 2 | 2 |
| miR397 | 0 | 0 | 3 |
| miR398 | 6 | 3 | 3 |
| miR437 | 1 | 0 | 1 |
| miR5049 | 33 | 64 | 30 |
| miR5062 | 4 | 4 | 5 |
| miR5067 | 0 | 0 | 2 |
| miR5070 | 3 | 2 | 0 |
| miR5086 | 1 | 1 | 0 |
| miR5174 | 4 | 2 | 5 |
| miR5175 | 5 | 1 | 1 |
| miR5180 | 5 | 4 | 1 |
| miR5181 | 11 | 17 | 6 |
| miR5200 | 15 | 0 | 3 |
| miR530 | 15 | 11 | 11 |
| miR5568 | 2 | 4 | 2 |
| miR6197 | 4 | 3 | 8 |
| miR9654 | 0 | 3 | 0 |
| miR9666 | 1 | 0 | 0 |
| miR9668 | 0 | 7 | 1 |
| miR9673 | 1 | 1 | 4 |
| miR9772 | 0 | 0 | 2 |
| miR9776 | 1 | 0 | 0 |
| miR9781 | 0 | 0 | 1 |
| miR9782 | 3 | 3 | 3 |
| miR9783 | 2 | 3 | 7 |

**Table S6.**
Gene targets of irrigated and drought stress-specific miRNAs.

| miRNA | Target Transcript ID | Functional Annotation |
|---|---|---|
| **Irrigated sample-specific miRNA targets** | | |
| miR1139-5p | TraesCS1B01G002300.1 | Disease-resistance protein (TIR-NBS-LRR class) |
| miR1139-5p | TraesCS1A01G006700.1 | Disease-resistance protein (TIR-NBS-LRR class) family |
| miR1139-5p | TraesCS1B01G002300.3 | NBS-LRR-like resistance protein |
| miR1139-5p | TraesCS1B01G002300.2 | Disease-resistance protein (TIR-NBS-LRR class) |
| miR9776-5p | TraesCS3B01G141300.1 | ATP-dependent RNA helicase |
| **Severe stress-specific miRNA targets** | | |
| miR397-5p | TraesCS1D01G044300.1 | Laccase |
| miR397-5p | TraesCS1A01G043900.1 | Laccase |
| miR397-5p | TraesCS1B01G056900.1 | Laccase |
| miR397-5p | TraesCS1D01G283000.1 | Laccase |
| miR397-5p | TraesCS1D01G044600.1 | Laccase |
| miR397-5p | TraesCS2D01G209400.1 | Laccase |
| miR397-5p | TraesCS3B01G393000.1 | Laccase |
| miR397-5p | TraesCS3D01G354400.1 | Laccase |
| miR397-5p | TraesCS3A01G360600.1 | Laccase |
| miR397-5p | TraesCS2B01G233500.1 | Laccase |
| miR397-5p | TraesCS2D01G209800.1 | Laccase |
| miR397-5p | TraesCS2B01G233700.1 | Laccase |
| miR9772-5p | TraesCS6D01G071900.1 | F-box family protein-like protein |
| miR9772-5p | TraesCS3B01G076500.1 | F-box family protein |
| miR9772-5p | TraesCS3A01G015900.1 | F-box family protein |
| miR9772-5p | TraesCS3D01G013200.1 | F-box family protein |
| miR397-3p | TraesCS5D01G435800.1 | Aldehyde oxidase, putative |
| miR397-3p | TraesCS5A01G427700.1 | Aldehyde oxidase, putative |
| miR397-5p | TraesCS4A01G096400.1 | Laccase |
| miR397-5p | TraesCS4D01G208900.1 | Laccase |
| miR9772-5p | TraesCS6D01G070800.1 | F-box domain containing protein, expressed |
| miR9772-5p | TraesCS6D01G070800.2 | F-box domain containing protein, expressed |
| miR9772-5p | TraesCS6D01G071500.2 | F-box family protein |
| miR9772-5p | TraesCS6A01G073200.1 | F-box family protein |
| miR9772-5p | TraesCS6B01G098200.1 | F-box family protein |
| miR9772-5p | TraesCS6D01G071500.1 | F-box family protein |
| miR9772-5p | TraesCSU01G133400.1 | F-box family protein |
| miR9772-5p | TraesCSU01G042700.1 | F-box family protein |
| miR9772-5p | TraesCSU01G217400.1 | F-box family protein |
| miR9772-5p | TraesCS4A01G375300.1 | Mitochondrial metalloendopeptidase OMA1 |
| miR9772-5p | TraesCS3B01G075900.1 | F-box family protein |
| miR397 | TraesCS7A01G047900.1 | Phosphatase 2C family protein, putative |
| miR397 | TraesCS5B01G377800.1 | Cysteine protease |
| **Targets of miRNAs common in irrigated and mild stress** | | |
| miR5200-3p | TraesCS3A01G001500.1 | Protein kinase |
| miR5200-3p | TraesCS3B01G000400.1 | Protein kinase |
| **Targets of miRNAs up-regulated in both stress conditions** | | |
| miR1122-3p | TraesCS4B01G160300.1 | Thiol:disulfide interchange protein txlA |
| miR1122-3p | TraesCS7D01G131200.1 | Disease-resistance protein (TIR-NBS-LRR class) family |

| | | |
|---|---|---|
| miR1122-3p | TraesCS7A01G005800.1 | Disease-resistance protein |
| miR1122-3p | TraesCS3D01G482500.1 | Crossover junction endonuclease mus81 |
| miR1122-3p | TraesCS5B01G444700.1 | Pseudouridine synthase |
| miR1122-3p | TraesCS5D01G448100.1 | Pseudouridine synthase |
| miR1122-3p | TraesCS5A01G441000.1 | Pseudouridine synthase |
| miR1122-3p | TraesCS5D01G539200.1 | Basic blue protein |
| miR1122-3p | TraesCS1B01G036500.1 | ERD (Early-responsive to dehydration stress) family protein |
| miR1122-3p | TraesCS3A01G258800.2 | Methyltransferase |
| miR1122-3p | TraesCS3D01G258900.1 | Methyltransferase |
| miR1122-3p | TraesCS3B01G291700.1 | Methyltransferase |
| miR1122-3p | TraesCS3A01G258800.1 | Methyltransferase |
| miR1122-3p | TraesCS6B01G297100.1 | F-box family protein |
| miR1122-3p | TraesCS6D01G245100.1 | F-box family protein |
| miR1130-3p | TraesCS7A01G005800.1 | Disease-resistance protein |
| miR1130-3p | TraesCS3D01G482500.1 | Crossover junction endonuclease mus81 |
| miR1130-3p | TraesCS2D01G248200.6 | Receptor protein kinase-like protein |
| miR1130-5p | TraesCS5D01G261800.1 | F-box family protein |
| miR1130-3p | TraesCS7D01G522300.1 | Pm3-like disease resistance protein |
| miR1137-3p | TraesCS5B01G557400.1 | Beta-fructofuranosidase, insoluble protein |
| miR1137-3p | TraesCS3B01G220700.1 | Eukaryotic translation initiation factor 6 |
| miR1137-3p | TraesCS3B01G220700.2 | Eukaryotic translation initiation factor 6 |
| miR1137-5p | TraesCS4B01G114400.1 | Anthranilate synthase alpha subunit 2, chloroplastic |
| miR1436-3p | TraesCS3D01G482500.1 | Crossover junction endonuclease mus81 |
| miR1436-3p | TraesCS2D01G072600.1 | Glycosyltransferase |
| miR1436-3p | TraesCS7A01G005800.1 | Disease resistance protein |
| miR1436-3p | TraesCS2D01G578800.1 | Shaggy-like protein kinase 41 |
| **Targets of miRNAs down-regulated in both stress conditions** | | |
| miR1127-3p | TraesCS2A01G360000.1 | Cysteine-rich receptor-like protein kinase 41 |
| miR1127-3p | TraesCS5B01G063100.1 | F-box family protein |
| miR1127-5p | TraesCS7D01G293800.2 | 65-kDa microtubule-associated protein 6 |
| miR1127-5p | TraesCS1D01G409900.1 | Beta-adaptin-like protein |
| miR1127-5p | TraesCS3B01G610700.1 | B3 domain-containing protein |
| miR1127-5p | TraesCS7D01G293800.1 | 65-kDa microtubule-associated-like protein |
| miR1127-5p | TraesCS6B01G449300.1 | Smad/FHA domain protein |
| miR1127-3p | TraesCS2B01G056900.1 | Histone acetyltransferase |
| miR1127-5p | TraesCS3A01G327800.1 | Haloacid dehalogenase superfamily protein |
| miR1127-3p | TraesCS3B01G251400.1 | StAR-related lipid transfer protein |
| miR1127-3p | TraesCS5B01G215200.1 | Glucan synthase-like 10 |
| miR1127-3p | TraesCS3B01G174800.1 | Protein kinase family protein |
| miR1127-3p | TraesCS6B01G256600.1 | RNA-binding protein |
| miR1127-3p | TraesCS5D01G023200.1 | Serine/threonine-protein kinase |
| miR1127-3p | TraesCS1B01G466000.1 | Leucine-rich repeat receptor-like protein kinase family protein |
| miR1127-5p | TraesCS6A01G380400.1 | DEAD/DEAH box RNA helicase family protein |
| miR1127-5p | TraesCS2A01G017400.1 | FACT complex subunit SPT16 |
| miR1127-5p | TraesCS5D01G261800.1 | F-box family protein |
| miR1127-5p | TraesCS1A01G065000.1 | Importin subunit beta-1 |
| miR1127-3p | TraesCS2D01G146600.1 | Gibberellin receptor GID1a |
| miR1127-5p | TraesCS2B01G452200.5 | Core-2/I-branching beta-1,6-N-acetylglucosaminyltransferase family protein |
| miR1127-3p | TraesCS1D01G409900.1 | Beta-adaptin-like protein |
| miR1127-3p | TraesCS5B01G451800.1 | Cactin |
| miR1127-3p | TraesCS4A01G492700.1 | Glutaredoxin family protein, expressed |
| miR1127-3p | TraesCSU01G156500.2 | 2-oxoglutarate (2OG) and Fe(II)-dependent oxygenase superfamily protein, putative |

| | | |
|---|---|---|
| miR1127-5p | TraesCS3A01G190300.12 | Exportin-7 |
| miR1127-5p | TraesCS3A01G190300.13 | Exportin-7 |
| miR1127-5p | TraesCS2D01G236200.6 | Transcription factor |
| miR1127-5p | TraesCS5B01G013700.1 | F-box protein |
| miR1127-5p | TraesCS2A01G131100.1 | Disease-resistance protein (TIR-NBS-LRR class) family |
| miR5175-5p | TraesCS2A01G485700.2 | Serine/threonine-protein kinase |
| miR5181-5p | TraesCS3B01G105100.1 | DUF674 family protein |

**Table S7.**

GO terms over-represented in miRNA targets which are unique to the severe stress samples. Universe consists of 1,516 target genes identified from the whole genome analysis (IWGSC 2018).

| GO term | Number of genes in Severe Stress | Number of genes in universe | ontology | GO Description | p_adjust | percentage |
|---|---|---|---|---|---|---|
| GO:0016722 | 14 | 14 | MF | oxidoreductase activity, oxidizing metal ions | 0 | 100 |
| GO:0046274 | 14 | 14 | BP | lignin catabolic process | 0 | 100 |
| GO:0052716 | 14 | 14 | MF | hydroquinone:oxygen oxidoreductase activity | 0 | 100 |
| GO:0005507 | 17 | 20 | MF | copper ion binding | 4.48E-09 | 85 |
| GO:0055114 | 29 | 65 | BP | oxidation-reduction process | 4.56E-07 | 44.62 |
| GO:0009698 | 13 | 15 | BP | phenylpropanoid metabolic process | 4.93E-07 | 86.67 |
| GO:0016682 | 9 | 9 | MF | oxidoreductase activity, acting on diphenols and related substances as donors, oxygen as acceptor | 1.78E-05 | 100 |
| GO:0009809 | 10 | 15 | BP | lignin biosynthetic process | 0.0015 | 66.67 |
| GO:0016491 | 19 | 48 | MF | oxidoreductase activity | 0.0018 | 39.58 |
| GO:0004601 | 9 | 14 | MF | peroxidase activity | 0.0042 | 64.29 |
| GO:0009832 | 9 | 14 | BP | plant-type cell wall biogenesis | 0.0068 | 64.29 |
| GO:0010413 | 8 | 11 | BP | glucuronoxylan metabolic process | 0.0070 | 72.73 |
| GO:0016209 | 8 | 12 | MF | antioxidant activity | 0.0075 | 66.67 |
| GO:0051920 | 8 | 12 | MF | peroxiredoxin activity | 0.0075 | 66.67 |
| GO:0009414 | 16 | 42 | BP | response to water deprivation | 0.0089 | 38.1 |
| GO:0009561 | 9 | 16 | BP | megagametogenesis | 0.0122 | 56.25 |
| GO:0030246 | 10 | 19 | MF | carbohydrate binding | 0.0122 | 52.63 |
| GO:0045492 | 8 | 13 | BP | xylan biosynthetic process | 0.0233 | 61.54 |

**Table S8.**
Enrichment of GO slim terms in SOM network modules 1 and 3.

| GO term | Number of genes in Network | Number of genes in universe | ontology | GO Description | p_adjust | percentage | Network Module |
|---|---|---|---|---|---|---|---|
| GO:0003676 | 211 | 441 | MF | nucleic acid binding | 2.99E-17 | 47.85 | 1 |
| GO:0006139 | 301 | 721 | BP | nucleobase-containing compound metabolic process | 4.07E-15 | 41.75 | 1 |
| GO:0006259 | 75 | 118 | BP | DNA metabolic process | 3.21E-13 | 63.56 | 1 |
| GO:0040029 | 48 | 64 | BP | regulation of gene expression, epigenetic | 2.70E-12 | 75 | 1 |
| GO:0003677 | 69 | 150 | MF | DNA binding | 0.00031 | 46 | 1 |
| GO:0007275 | 305 | 897 | BP | multicellular organism development | 0.003297 | 34 | 1 |
| GO:0007049 | 64 | 149 | BP | cell cycle | 0.006148 | 42.95 | 1 |
| GO:0009791 | 220 | 642 | BP | post-embryonic development | 0.026352 | 34.27 | 1 |
| GO:0009058 | 381 | 1179 | BP | biosynthetic process | 0.033668 | 32.32 | 1 |
| GO:0003723 | 26 | 52 | MF | RNA binding | 0.036492 | 50 | 1 |
| GO:0016043 | 252 | 753 | BP | cellular component organization | 0.036492 | 33.47 | 1 |
| GO:0016301 | 262 | 443 | MF | kinase activity | 3.51E-27 | 59.14 | 3 |
| GO:0009605 | 470 | 1003 | BP | response to external stimulus | 7.32E-20 | 46.86 | 3 |
| GO:0009607 | 395 | 815 | BP | response to biotic stimulus | 5.43E-19 | 48.47 | 3 |
| GO:0007154 | 442 | 939 | BP | cell communication | 6.07E-19 | 47.07 | 3 |
| GO:0004872 | 138 | 212 | MF | receptor activity | 1.42E-18 | 65.09 | 3 |
| GO:0004871 | 141 | 227 | MF | signal transducer activity | 2.16E-16 | 62.11 | 3 |
| GO:0008219 | 185 | 336 | BP | cell death | 3.10E-14 | 55.06 | 3 |
| GO:0006464 | 317 | 667 | BP | cellular protein modification process | 4.13E-13 | 47.53 | 3 |
| GO:0000166 | 339 | 728 | MF | nucleotide binding | 1.08E-12 | 46.57 | 3 |
| GO:0007165 | 340 | 748 | BP | signal transduction | 7.52E-11 | 45.45 | 3 |
| GO:0009991 | 131 | 242 | BP | response to extracellular stimulus | 2.76E-09 | 54.13 | 3 |
| GO:0019538 | 364 | 833 | BP | protein metabolic process | 5.36E-09 | 43.7 | 3 |
| GO:0003824 | 663 | 1677 | MF | catalytic activity | 1.62E-08 | 39.53 | 3 |
| GO:0006810 | 331 | 751 | BP | transport | 1.68E-08 | 44.07 | 3 |
| GO:0009719 | 363 | 843 | BP | response to endogenous stimulus | 5.22E-08 | 43.06 | 3 |
| GO:0016740 | 360 | 837 | MF | transferase activity | 5.22E-08 | 43.01 | 3 |
| GO:0030246 | 46 | 66 | MF | carbohydrate binding | 1.30E-07 | 69.7 | 3 |
| GO:0009856 | 90 | 159 | BP | pollination | 2.26E-07 | 56.6 | 3 |
| GO:0005215 | 162 | 338 | MF | transporter activity | 1.34E-06 | 47.93 | 3 |
| GO:0009875 | 27 | 34 | BP | pollen-pistil interaction | 2.65E-06 | 79.41 | 3 |
| GO:0009838 | 29 | 44 | BP | abscission | 0.000472 | 65.91 | 3 |
| GO:0005515 | 222 | 522 | MF | protein binding | 0.00071 | 42.53 | 3 |
| GO:0006950 | 620 | 1632 | BP | response to stress | 0.001046 | 37.99 | 3 |
| GO:0005102 | 29 | 49 | MF | receptor binding | 0.007335 | 59.18 | 3 |

**Table S9.**

Publicly available flag leaf and drought experiment RNA-Seq samples (www.wheat-expression.com; Borill et al. 2016) included for WGCNA analysis.

| | *Age* | *Stress-disease* | *Phenotype code* | *Sample IDs* |
|---|---|---|---|---|
| (1) | Anther differentiation stage | Early drought | AD_S | SRR5383650, SRR5383900, SRR5383901 |
| | | None | AD_C | SRR5383933, SRR5383934, SRR5383935 |
| | Tetrad stage | Late drought | T_S | SRR5383936, SRR5383938, SRR5383941 |
| | | None | T_C | SRR5383944, SRR5383945, SRR5383947 |
| (2) | *Age* | *Stress-disease* | *Phenotype code* | *Sample IDs* |
| | 7 days | 6 hour PEG stress | PEG6 | SRR1542409, SRR1542408 |
| | | 1 hour PEG stress | PEG1 | SRR1542407, SRR1542406 |
| | | None | IS | SRR1542405, SRR1542404 |
| (3) | *Age* | *Stress-disease* | *Phenotype code* | *Sample IDs* |
| | Flag leaf stage | None | IP | Sample_61A, Sample_62A, Sample_63A, Sample_75A, Sample_77A, Sample_78A |
| | Full boot | | | Sample_94B, Sample_95A, Sample_96A |
| | 30% spike | | | Sample_109A, Sample_110A, Sample_111B |
| | Ear emergence | | | Sample_127A, Sample_128A, Sample_129A |
| | Anthesis | | | Sample_166A, Sample_167A, Sample_168A |
| | Milk grain stage | | | Sample_175A, Sample_176A, Sample_177A |
| | Dough | | | Sample_202A, Sample_203A, Sample_204A |
| | Ripening | | | Sample_223B, Sample_225A, Sample_224A |
| (4) | *Age* | *Stress-disease* | *Phenotype code* | *Sample IDs* |
| | 12 dpa | None | IP | SRR085459, SRR085458, SRR085457 |
| (5) | *Age* | *Stress-disease* | *Phenotype code* | *Sample IDs* |
| | 30 dpa | None | IP | SRR3068482 |
| | 25 dpa | | | SRR3068481 |
| | 15 dpa | | | SRR3068477 |
| | anthesis | | | SRR3068439 |
| | Ear emergence | | | SRR3068387 |

(1) Ma et al. 2017
(2) Liu et al. 2017
(3), (4), (5) Ramírez-González et al. 2018

**Table S10.**
CoDReGs' SOM module classification and annotation.

| | |
|---|---|
| **Module 1** | |
| CoDReG_6_011_01 | Acid beta-fructofuranosidase |
| CoDReG_6_581_01 | Dehydrin |
| CoDReG_6_593_01 | Serine-rich protein |
| CoDReG_1_544_01 | Late embryogenesis abundant protein |
| CoDReG_6_005_01 | Leucine-rich repeat receptor-like protein kinase family protein |
| CoDReG_6_559_01 | 1-Aminocyclopropane-1-carboxylate oxidase |
| CoDReG_2_735_01 | Isoaspartyl peptidase/L-asparaginase |
| **Module 2** | |
| CoDReG_3_608_01 | Laccase |
| CoDReG_3_508_01 | Glycosyltransferase |
| CoDReG_3_438_01 | GDSL esterase/lipase |
| CoDReG_6_562_01 | Auxin-responsive family protein |
| CoDReG_1_549_01 | Lipid transfer protein |
| **Module 3** | |
| CoDReG_2_590_01 | Chitinase |
| CoDReG_1_002_01 | Receptor protein kinase, putative |
| CoDReG_7_567_01 | Glutamine dumper, putative |
| CoDReG_1_578_01 | Receptor-like protein kinase, putative, expressed |
| CoDReG_1_582_01 | ATP-dependent zinc metalloprotease FtsH |
| CoDReG_1_009_01 | Receptor-like kinase |
| CoDReG_1_464_01 | Transmembrane protein, putative (DUF1218) |
| CoDReG_2_734_01 | Nuclease |
| CoDReG_2_702_01 | Senescence-associated family protein (DUF581) |
| CoDReG_3_712_01 | Beta-1,3-glucanase |
| CoDReG_5_569_01 | Protein DETOXIFICATION |
| CoDReG_3_719_01 | Protein kinase |
| CoDReG_1_374_01 | Expansin protein |
| CoDReG_3_026_01 | Receptor-like kinase |
| CoDReG_5_513_01 | Glycosyltransferase |
| CoDReG_1_533_01 | Glycosyltransferase |
| CoDReG_3_724_01 | Protein DETOXIFICATION |
| CoDReG_3_644_01 | DUF1685 family protein |
| CoDReG_7_721_01 | Expansin-like protein |
| CoDReG_5_046_01 | Kinase, putative |
| CoDReG_2_012_01 | 2-oxoglutarate (2OG) and Fe(II)-dependent oxygenase superfamily protein |
| CoDReG_6_061_01 | Chlorophyll a-b binding protein, chloroplastic |
| CoDReG_2_194_01 | Cellulose synthase-like protein |
| CoDReG_3_024_01 | Trypsin inhibitor |
| CoDReG_4_456_01 | Germin-like protein |
| CoDReG_2_718_01 | Anthocyanidin reductase |
| **Module 5** | |
| CoDReG_2_624_01 | Phenylalanine ammonia-lyase |
| CoDReG_7_619_01 | Xyloglucan endotransglucosylase/hydrolase |
| CoDReG_6_023_01 | Peroxidase |
| CoDReG_6_592_01 | Embryogenesis transmembrane protein-like |
| CoDReG_5_503_02 | Beta-glucosidase |
| CoDReG_1_337_01 | Glutathione S-transferase |
| CoDReG_7_182_01 | Embryogenesis transmembrane protein |
| CoDReG_2_751_01 | Heavy metal transport/detoxification superfamily protein |
| CoDReG_2_572_01 | Amino acid transporter, putative |
| CoDReG_2_667_01 | Glycosyltransferase |
| CoDReG_2_765_01 | NAC domain-containing protein, putative |
| CoDReG_4_678_01 | Arabinogalactan protein 5 |
| CoDReG_7_694_01 | Protein kinase |

**Table S11.**

CoDReG genes synteny with rice. Only the HC genes in the A genome are shown. Start pos.: Gene starting position at the RefSeqv1 pseudomolecule (IWGSC 2018); Chr: chromosome.

| | *Triticum aestivum* | | | *Oryza sativa* | | |
|---|---|---|---|---|---|---|
| CoDReG | A genome gene model | Start pos. | Chr | Gene | Start pos. | Chr |
| CoDReG_1A_337_01 | TraesCS1A01G186100 | 337,300,913 | 1A | OS10T0530900-01 | 20,633,891 | os10 |
| | TraesCS1A01G186200 | 337,629,038 | 1A | OS10T0530200-01 | 20,610,080 | os10 |
| | TraesCS1A01G186300 | 337,654,251 | 1A | OS10T0529400-01 | 20,592,565 | os10 |
| | TraesCS1A01G186400 | 337,655,620 | 1A | OS10T0529300-01 | 20,589,767 | os10 |
| | TraesCS1A01G186500 | 337,657,719 | 1A | | | |
| | TraesCS1A01G186600 | 337,677,617 | 1A | OS10T0528300-01 | 20,556,396 | os10 |
| | TraesCS1A01G186700 | 337,703,251 | 1A | OS10T0527800-01 | 20,498,004 | os10 |
| | TraesCS1A01G186800 | 337,776,142 | 1A | | | |
| | TraesCS1A01G186900 | 337,781,454 | 1A | | | |
| CoDReG_1A_374_01 | TraesCS1A01G212000 | 374,689,661 | 1A | | | |
| | TraesCS1A01G212200 | 374,958,632 | 1A | | | |
| | TraesCS1A01G212300 | 375,015,072 | 1A | | | |
| | TraesCS1A01G212400 | 375,112,335 | 1A | OS10T0555900-01 | 21,847,853 | os10 |
| | TraesCS1A01G212600 | 375,467,613 | 1A | | | |
| CoDReG_1A_464_01 | TraesCS1A01G270900 | 464,901,178 | 1A | OS05T0433400-00 | 21,240,878 | os5 |
| | TraesCS1A01G271000 | 464,911,436 | 1A | | | |
| | TraesCS1A01G271100 | 465,074,275 | 1A | OS05T0433800-00 | 21,258,792 | os5 |
| | TraesCS1A01G271300 | 465,105,200 | 1A | | | |
| | TraesCS1A01G271400 | 465,128,941 | 1A | | | |
| | TraesCS1A01G271700 | 465,282,112 | 1A | | | |
| CoDReG_1A_533_01 | TraesCS1A01G347100 | 533,253,006 | 1A | | | |
| | TraesCS1A01G347200 | 533,257,093 | 1A | OS01T0736100-01 | 30,694,998 | os1 |
| | TraesCS1A01G347400 | 533,323,409 | 1A | | | |
| | TraesCS1A01G347500 | 533,409,211 | 1A | | | |
| | TraesCS1A01G347600 | 533,445,241 | 1A | | | |
| CoDReG_2A_590_01 | TraesCS2A01G350400 | 590,689,950 | 2A | | | |
| | TraesCS2A01G350500 | 590,719,668 | 2A | | | |
| | TraesCS2A01G350600 | 590,725,795 | 2A | | | |
| | TraesCS2A01G350700 | 590,816,875 | 2A | OS04T0493400-01 | 24,687,753 | os4 |
| | TraesCS2A01G350800 | 590,965,353 | 2A | | | |
| | TraesCS2A01G350900 | 590,973,934 | 2A | | | |
| CoDReG_2A_624_01 | TraesCS2A01G380800 | 624,359,354 | 2A | | | |
| | TraesCS2A01G380900 | 624,394,333 | 2A | | | |
| | TraesCS2A01G381000 | 624,446,921 | 2A | | | |
| | TraesCS2A01G381100 | 624,458,285 | 2A | | | |
| CoDReG_2A_667_01 | TraesCS2A01G410000 | 667,942,211 | 2A | OS04T0556400-01 | 27,843,255 | os4 |
| | TraesCS2A01G410100 | 667,959,454 | 2A | | | |
| | TraesCS2A01G410200 | 667,964,272 | 2A | | | |
| | TraesCS2A01G410300 | 668,011,891 | 2A | | | |
| CoDReG_2A_702_01 | TraesCS2A01G453400 | 702,937,263 | 2A | | | |
| | TraesCS2A01G453500 | 702,951,018 | 2A | | | |
| | TraesCS2A01G453600 | 702,956,579 | 2A | | | |
| | TraesCS2A01G453700 | 703,095,397 | 2A | OS02T0686800-01 | 28,136,347 | os2 |
| | TraesCS2A01G453800 | 703,105,806 | 2A | | | |
| | TraesCS2A01G453900 | 703,111,013 | 2A | | | |
| | TraesCS2A01G454000 | 703,123,052 | 2A | | | |
| | TraesCS2A01G454200 | 703,282,420 | 2A | OS04T0586000-00 | 29,610,157 | os4 |
| | TraesCS2A01G454300 | 703,291,243 | 2A | | | |
| | TraesCS2A01G454400 | 703,346,045 | 2A | | | |
| | TraesCS2A01G454500 | 703,382,028 | 2A | | | |
| | TraesCS2A01G454600 | 703,402,870 | 2A | | | |
| | TraesCS2A01G454700 | 703,448,159 | 2A | | | |
| | TraesCS2A01G454800 | 703,545,719 | 2A | OS04T0585900-01 | 29,606,210 | os4 |
| CoDReG_2A_718_01 | TraesCS2A01G481200 | 718,574,651 | 2A | OS04T0630300-01 | 32,059,560 | os4 |
| | TraesCS2A01G481300 | 718,622,056 | 2A | | | |
| | TraesCS2A01G481400 | 718,722,290 | 2A | | | |
| | TraesCS2A01G481500 | 718,728,983 | 2A | | | |
| | TraesCS2A01G481700 | 718,818,748 | 2A | OS04T0630900-01 | 32,090,880 | os4 |
| | TraesCS2A01G481800 | 718,835,431 | 2A | | | |
| | TraesCS2A01G481900 | 718,837,481 | 2A | | | |
| | TraesCS2A01G482100 | 718,883,587 | 2A | | | |
| | TraesCS2A01G482200 | 718,916,822 | 2A | | | |
| | TraesCS2A01G482300 | 718,929,125 | 2A | | | |
| | TraesCS2A01G483100 | 719,218,058 | 2A | | | |
| CoDReG_2A_734_01 | TraesCS2A01G506800 | 734,524,488 | 2A | | | |
| | TraesCS2A01G507100 | 734,621,298 | 2A | | | |
| | TraesCS2A01G507300 | 734,629,406 | 2A | OS04T0652700-01 | 33,253,920 | os4 |
| | TraesCS2A01G507400 | 734,634,074 | 2A | | | |
| | TraesCS2A01G507500 | 734,643,084 | 2A | | | |
| | TraesCS2A01G507600 | 734,647,130 | 2A | | | |
| | TraesCS2A01G507800 | 734,739,197 | 2A | | | |
| CoDReG_2A_735_01 | TraesCS2A01G510400 | 735,600,166 | 2A | OS04T0650700-01 | 33,161,539 | os4 |
| | TraesCS2A01G510700 | 735,701,087 | 2A | | | |
| | TraesCS2A01G510800 | 735,761,447 | 2A | | | |
| CoDReG_2A_751_01 | TraesCS2A01G538900 | 751,526,265 | 2A | | | |
| | TraesCS2A01G539000 | 751,546,620 | 2A | | | |
| | TraesCS2A01G539100 | 751,552,937 | 2A | | | |
| | TraesCS2A01G539300 | 751,662,481 | 2A | | | |
| | TraesCS2A01G539400 | 751,844,741 | 2A | | | |
| | TraesCS2A01G539500 | 751,892,375 | 2A | | | |
| | TraesCS2A01G539600 | 752,048,019 | 2A | | | |
| CoDReG_2A_765_01 | TraesCS2A01G565900 | 765,277,860 | 2A | | | |
| | TraesCS2A01G566000 | 765,373,519 | 2A | | | |
| | TraesCS2A01G566100 | 765,392,440 | 2A | | | |
| | TraesCS2A01G566200 | 765,441,104 | 2A | | | |
| | TraesCS2A01G566300 | 765,514,989 | 2A | | | |
| | TraesCS2A01G566400 | 765,546,770 | 2A | | | |
| CoDReG_3A_438_01 | TraesCS3A01G234900 | 438,603,930 | 3A | OS01T0649400-00 | 26,202,814 | os1 |
| | TraesCS3A01G235000 | 438,611,841 | 3A | | | |
| | TraesCS3A01G235100 | 438,637,095 | 3A | OS01T0650900-00 | 26,286,441 | os1 |
| | TraesCS3A01G235500 | 439,277,465 | 3A | | | |
| CoDReG_3A_508_01 | TraesCS3A01G279100 | 508,520,011 | 3A | | | |
| | TraesCS3A01G279500 | 508,541,937 | 3A | OS01T0735900-01 | 30,689,850 | os1 |
| | TraesCS3A01G279600 | 508,922,280 | 3A | OS05T0526900-01 | 26,196,244 | os5 |
| | TraesCS3A01G279700 | 509,020,231 | 3A | OS05T0527000-01 | 26,198,411 | os5 |
| | TraesCS3A01G279800 | 509,250,162 | 3A | | | |
| | TraesCS3A01G280000 | 509,375,867 | 3A | | | |
| CoDReG_3A_608_01 | TraesCS3A01G360400 | 608,194,379 | 3A | | | |
| | TraesCS3A01G360600 | 608,346,162 | 3A | | | |
| | TraesCS3A01G361100 | 608,750,119 | 3A | | | |
| CoDReG_3A_644_01 | TraesCS3A01G396800 | 644,608,921 | 3A | OS01T0898800-00 | 39,079,600 | os1 |
| | TraesCS3A01G396900 | 644,622,945 | 3A | | | |
| | TraesCS3A01G397000 | 644,666,540 | 3A | | | |
| CoDReG_3A_712_01 | TraesCS3A01G481500 | 712,545,178 | 3A | OS01T0940800-01 | 41,349,731 | os1 |
| | TraesCS3A01G482200 | 712,728,393 | 3A | | | |
| | TraesCS3A01G483000 | 713,534,548 | 3A | OS01T0944700-01 | 41,533,329 | os1 |
| CoDReG_3A_719_01 | TraesCS3A01G493100 | 719,775,905 | 3A | | | |
| | TraesCS3A01G493300 | 719,835,735 | 3A | | | |
| | TraesCS3A01G493500 | 719,933,035 | 3A | | | |
| | TraesCS3A01G493700 | 719,991,629 | 3A | | | |
| | TraesCS3A01G493800 | 720,075,369 | 3A | | | |
| | TraesCS3A01G493900 | 720,214,469 | 3A | | | |
| | TraesCS3A01G494000 | 720,228,117 | 3A | | | |
| CoDReG_3A_724_01 | TraesCS3A01G499200 | 724,482,590 | 3A | | | |
| | TraesCS3A01G499300 | 724,491,445 | 3A | | | |
| | TraesCS3A01G499800 | 724,591,000 | 3A | | | |
| | TraesCS3A01G500000 | 724,645,493 | 3A | | | |
| CoDReG_4A_678_01 | TraesCS4A01G404800 | 678,340,392 | 4A | | | |
| | TraesCS4A01G405100 | 678,373,388 | 4A | | | |
| | TraesCS4A01G405300 | 678,375,972 | 4A | | | |
| | TraesCS4A01G405500 | 678,408,482 | 4A | | | |
| | TraesCS4A01G405600 | 678,418,867 | 4A | | | |
| CoDReG_5A_046_01 | TraesCS5A01G051100 | 46,494,698 | 5A | | | |
| | TraesCS5A01G051200 | 46,500,373 | 5A | | | |
| | TraesCS5A01G051700 | 46,708,702 | 5A | | | |
| CoDReG_5A_503_02 | TraesCS5A01G295100 | 503,776,182 | 5A | OS09T0511600-01 | 19,882,716 | os9 |
| | TraesCS5A01G295200 | 503,783,117 | 5A | | | |
| | TraesCS5A01G295300 | 503,795,009 | 5A | | | |
| | TraesCS5A01G295400 | 503,872,199 | 5A | | | |
| | TraesCS5A01G295500 | 503,927,180 | 5A | | | |
| | TraesCS5A01G295600 | 503,930,866 | 5A | | | |
| CoDReG_5A_513_01 | TraesCS5A01G304900 | 513,868,622 | 5A | OS09T0517900-01 | 20,196,707 | os9 |
| | TraesCS5A01G305000 | 513,959,483 | 5A | | | |
| | TraesCS5A01G305100 | 514,003,177 | 5A | | | |
| CoDReG_5A_569_01 | TraesCS5A01G368500 | 569,151,700 | 5A | | | |
| | TraesCS5A01G368600 | 569,181,086 | 5A | | | |
| | TraesCS5A01G368700 | 569,217,518 | 5A | | | |
| | TraesCS5A01G368800 | 569,222,678 | 5A | | | |
| CoDReG_7A_567_01 | TraesCS7A01G392000 | 567,792,293 | 7A | OS06T0633100-01 | 25,655,524 | os6 |
| | TraesCS7A01G392100 | 568,129,834 | 7A | | | |
| | TraesCS7A01G392200 | 568,579,817 | 7A | | | |
| | TraesCS7A01G392300 | 568,695,329 | 7A | | | |
| CoDReG_7A_619_01 | TraesCS7A01G426100 | 619,165,697 | 7A | OS06T0696400-01 | 29,133,106 | os6 |
| | TraesCS7A01G426600 | 619,575,419 | 7A | | | |
| | TraesCS7A01G426700 | 619,632,998 | 7A | | | |
| | TraesCS7A01G426900 | 619,907,022 | 7A | | | |
| | TraesCS7A01G427000 | 620,171,467 | 7A | | | |
| | TraesCS7A01G427100 | 620,370,075 | 7A | | | |
| | TraesCS7A01G427200 | 620,411,760 | 7A | | | |
| | TraesCS7A01G427300 | 620,487,009 | 7A | | | |
| | TraesCS7A01G427600 | 620,749,363 | 7A | | | |
| CoDReG_7A_696_01 | TraesCS7A01G507100 | 694,196,451 | 7A | | | |
| | TraesCS7A01G507200 | 694,252,172 | 7A | | | |
| | TraesCS7A01G507400 | 694,329,927 | 7A | | | |
| | TraesCS7A01G507500 | 694,356,044 | 7A | | | |
| | TraesCS7A01G507600 | 694,373,607 | 7A | | | |
| | TraesCS7A01G507700 | 694,420,331 | 7A | | | |

**Table S12.**

CoDReGs with syntenic positions with rice drought tolerance QTLs. Rice chromosomal positions (IRGSP 2017) are indicated. *: from the Q-TARO database (Yonemaru 2010)

| CoDReG | Trait* | Trait category 1* | Trait category 2* | Chr | Start pos. | End pos. |
|---|---|---|---|---|---|---|
| CoDReG_1A_337_01 | osmotic adjustment | Resistance or Tolerance | Drought tolerance | os10 | 21,384,897 | 21,932,522 |
| CoDReG_1A_374_01 | dry weight per plant | Resistance or Tolerance | Blast resistance | os10 | 21,624,625 | 21,629,868 |
| | Percent sterility | Resistance or Tolerance | Drought tolerance | os10 | 21,474,732 | 21,503,768 |
| CoDReG_1A_464_01 | Salt-soluble storage proteins | Physiological trait | Eating quality | os5 | 22,309,016 | 25,730,022 |
| | panicle or tiller no.per m2 | Resistance or Tolerance | Drought tolerance | os5 | 22,309,016 | 24,371,958 |
| CoDReG_1A_533_01 | dwarf phenotype and leaves with abnormally rounded tip regions | Morphological trait | Dwarf | os1 | 29,747,390 | 29,878,594 |
| | leaf rolling of culturing (LRC) | Resistance or Tolerance | Drought tolerance | os1 | 28,571,547 | 34,744,337 |
| CoDReG_2A_590_01 | Plant age in leaf number F-C | Morphological trait | Culm/leaf | os4 | 24,646,179 | 25,619,040 |
| | root fresh weight | Resistance or Tolerance | Drought tolerance | os4 | 25,626,203 | 33,083,265 |
| CoDReG_2A_624_01 | root fresh weight | Resistance or Tolerance | Drought tolerance | os4 | 25,626,203 | 33,083,265 |
| CoDReG_2A_667_01 | Maximum new root length Average | Morphological trait | Root | os4 | 27,941,367 | 30,762,314 |
| | root fresh weight | Resistance or Tolerance | Drought tolerance | os4 | 25,626,203 | 33,083,265 |
| CoDReG_2A_702_01 | bacterial blight resistance | Resistance or Tolerance | Bacterial blight resistance | os4 | 29,644,621 | 33,083,265 |
| | panicle number per hill | Resistance or Tolerance | Drought tolerance | os4 | 29,644,621 | 31,356,186 |
| CoDReG_2A_718_01 | bacterial blight resistance | Resistance or Tolerance | Bacterial blight resistance | os4 | 32,036,440 | 32,061,259 |
| | Plant height-control | Resistance or Tolerance | Drought tolerance | os4 | 31,648,882 | 32,246,967 |
| CoDReG_2A_734_01 | Basal root thickness | Resistance or Tolerance | Drought tolerance | os4 | 33,234,004 | 33,430,563 |
| CoDReG_2A_735_01 | Basal root thickness | Resistance or Tolerance | Drought tolerance | os4 | 33,234,004 | 33,430,563 |
| CoDReG_2A_751_01 | leaf drying | Resistance or Tolerance | Drought tolerance | os4 | 34,247,154 | 35,283,358 |
| CoDReG_2A_765_01 | Shoot weight low N condition | Resistance or Tolerance | Other soil stress tolerance | os4 | 35,277,669 | 35,283,358 |
| | Biomass-control | Resistance or Tolerance | Drought tolerance | os4 | 35,277,669 | 35,283,358 |
| CoDReG_3A_438_01 | initial plant height | Resistance or Tolerance | Submergency tolerance | os1 | 26,419,476 | 29,360,353 |
| | leaf rolling of culturing (LRC) | Resistance or Tolerance | Drought tolerance | os1 | 28,571,547 | 34,744,337 |
| CoDReG_3A_508_01 | dwarf phenotype and leaves with abnormally rounded tip regions | Morphological trait | Dwarf | os1 | 29,747,390 | 29,878,594 |
| | leaf rolling of culturing (LRC) | Resistance or Tolerance | Drought tolerance | os1 | 28,571,547 | 34,744,337 |
| CoDReG_3A_608_01 | Yield(1998) | Morphological trait | Seed | os1 | 36,226,902 | 39,470,731 |
| | Plant height | Resistance or Tolerance | Drought tolerance | os1 | 36,697,294 | 42,364,623 |
| CoDReG_3A_644_01 | cold tolerance at seedling stage (CTS) | Resistance or Tolerance | Cold tolerance | os1 | 38,490,966 | 39,646,396 |
| | Plant height (Low N level, Soil culture , Maturity ) | Resistance or Tolerance | Other soil stress tolerance | os1 | 36,697,294 | 42,364,623 |
| CoDReG_3A_712_01 | Tiller number | Morphological trait | Culm/leaf | os1 | 41,444,285 | 42,364,623 |
| | leaf drying score | Resistance or Tolerance | Drought tolerance | os1 | 41,444,285 | 44,211,418 |
| CoDReG_3A_719_01 | panicle length | Morphological trait | Panicle/flower | os1 | 42,157,734 | 42,364,623 |
| | Plant height | Resistance or Tolerance | Drought tolerance | os1 | 41,923,730 | 42,364,623 |
| CoDReG_3A_724_01 | panicle length | Morphological trait | Panicle/flower | os1 | 42,157,734 | 42,364,623 |
| | Plant height | Resistance or Tolerance | Drought tolerance | os1 | 41,923,730 | 42,364,623 |
| CoDReG_4A_678_01 | Plant height | Morphological trait | Dwarf | os3 | 5,210,684 | 5,584,862 |
| | lateral root number | Resistance or Tolerance | Drought tolerance | os3 | 5,758,005 | 5,758,498 |
| CoDReG_5A_046_01 | Lowest elongated internode | Resistance or Tolerance | Submergency tolerance | os12 | 25,203,610 | 26,368,115 |
| | Drought score | Resistance or Tolerance | Drought tolerance | os12 | 26,277,931 | 27,748,876 |
| CoDReG_5A_503_02 | ABA accumulation | Resistance or Tolerance | Drought tolerance | os9 | 19,559,036 | 19,562,133 |
| | maximum root length (non stress) | Resistance or Tolerance | Drought tolerance | os9 | 20,078,749 | 20,828,859 |
| CoDReG_5A_513_01 | maximum root length (non stress) | Resistance or Tolerance | Drought tolerance | os9 | 20,078,749 | 20,828,859 |
| CoDReG_5A_569_01 | Elongation of plant height | Morphological trait | Dwarf | os9 | 22,688,515 | 22,690,613 |
| | Days to heading-control | Resistance or Tolerance | Drought tolerance | os9 | 21,313,734 | 22,021,406 |
| CoDReG_7A_567_01 | 3rd internode length | Morphological trait | Dwarf | os6 | 25,794,136 | 25,797,291 |
| | Penetrated root thickness | Resistance or Tolerance | Drought tolerance | os6 | 23,740,405 | 25,797,291 |
| CoDReG_7A_619_01 | Carbon isotope discrimination ˘ | Physiological trait | Source activity | os6 | 29,477,181 | 29,477,319 |
| | Panicles per plant | Resistance or Tolerance | Drought tolerance | os6 | 26,569,436 | 28,130,383 |
| CoDReG_7A_696_01 | Panicles per plant | Resistance or Tolerance | Drought tolerance | os6 | 26,569,436 | 28,130,383 |

**Table S13.**

Unbalanced triads enrichment in CoDReGs in irrigated (I), medium stress (MS) and severe stress (SS) levels.

| | I | | MS | | SS | |
|---|---|---|---|---|---|---|
| | *All triads* | *CoDReGs* | *All triads* | *CoDReGs* | *All triads* | *CoDReGs* |
| *Unbalanced* | 4468 | 70 | 4586 | 80 | 4565 | 77 |
| *Balanced* | 9008 | 29 | 9217 | 21 | 8856 | 19 |
| | | P=1e-5 | | P=1e-5 | | P=1e-5 |

P: Significant exact Fisher test P values are indicated for significant tests. Triads enrichment is shown with a blue cell. For all triads, genes with TPM>0.5 have been considered. For CoDReGs, genes with known TPM (TPM>0) have been considered.

**Table S14.**

Proposed nomenclature for RefSeqv1 dehydrin gene models

| RefSeqv1 ID | Proposed name | |
|---|---|---|
| TraesCS3A01G254600 | **DHN35** | *DHN35-A1* |
| TraesCS3B01G286600 | | *DHN35-B1* |
| TraesCS3D01G255500 | | *DHN35-D1* |
| TraesCS3A01G396200 | **DHN33** | *DHN33-A1* |
| TraesCS3B01G428200 | | *DHN33-B1* |
| TraesCS3D01G390200 | | *DHN33-D1* |
| TraesCS4A01G250900 | **DHN6** | *DHN6-A1* |
| TraesCS4B01G064200 | | *DHN6-B1* |
| TraesCS4D01G063100 | | *DHN6-D1* |
| TraesCS4A01G455000LC | **DHN36.2** | *DHN36-A2* |
| TraesCS4A01G299100 | **DHN36** | *DHN36-A1* |
| TraesCS4B01G014300 | | *DHN36-B1* |
| TraesCS4D01G012500 | | *DHN36-D1* |
| TraesCS5A01G369800 | **DHN2** | *DHN2-A1* |
| TraesCS5B01G372100 | | *DHN2-B1* |
| TraesCS5D01G379200 | | *DHN2-D1* |
| TraesCS5A01G369900 | **DHN1** | *DHN1-A1* |
| TraesCS5B01G372200 | | *DHN1-B1* |
| TraesCS5D01G379300 | | *DHN1-D1* |
| TraesCS5A01G424700 | **DHN38** | *DHN38-A1* |
| TraesCS5A01G424800 | | *DHN38-A2* |
| TraesCS5B01G426700 | | *DHN38-B1* |
| TraesCS5B01G426800 | | *DHN38-B2* |
| TraesCS5D01G433200 | | *DHN38-D1* |
| TraesCS5D01G433300 | | *DHN38-D2* |
| TraesCS5D01G519300LC | **DHN38.3** | *DHN38-D3* |
| TraesCS6A01G059800 | **DHN37** | *DHN37-A1* |
| TraesCSU01G086200 | | *DHN37-B1* |
| TraesCSU01G122200 | | *DHN37-D1* |
| TraesCS6A01G253300 | **DHN11** | *DHN11-A1* |
| TraesCS6B01G273400 | | *DHN11-B1* |
| TraesCS6D01G234700 | | *DHN11-D1* |
| TraesCS6A01G350100 | **DHN5.1** | *DHN5-A1* |
| TraesCS6D01G332500 | | *DHN5-D1* |
| TraesCS6A01G350200 | **DHN9.6** | *DHN9-A1* |
| TraesCS6D01G332600 | | *DHN9-D1* |
| TraesCS6A01G350300 | **DHN5.2** | *DHN5-A2* |
| TraesCS6D01G332700 | | *DHN5-D2* |
| TraesCS6B01G383200 | **DHN5.3** | *DHN5-B3* |
| TraesCS6B01G695200LC | **DHN5.4** | *DHN5-B4* |
| TraesCS6A01G350500 | **DHN4** | *DHN4-A1* |
| TraesCS6B01G383500 | | *DHN4-B1* |
| TraesCS6D01G332900 | | *DHN4-D1* |
| TraesCS6A01G350600 | **DHN3.1** | *DHN3-A1* |
| TraesCS6B01G695700LC | | *DHN3-B1* |
| TraesCS6B01G695800LC | | *DHN3-B2* |
| TraesCS6D01G333000 | | *DHN3-D1* |
| TraesCS6B01G695900LC | **DHN3.3** | *DHN3-B3* |
| TraesCS6D01G333100 | **DHN3.4** | *DHN3-D4* |
| TraesCS6A01G350800 | **DHN3.5** | *DHN3-A5* |
| TraesCS6A01G350700 | **DHN3.6** | *DHN3-A6* |
| TraesCS6B01G383600 | | *DHN3-B6* |
| TraesCS6D01G333200 | | *DHN3-D6* |
| TraesCS6A01G350900 | **DHN3.7** | *DHN3-A7* |
| TraesCS6D01G333300 | **DHN3.8** | *DHN3-D8* |
| TraesCS6B01G383800 | **DHN3.9** | *DHN3-B9* |
| TraesCS6D01G333600 | | *DHN3-D9* |
| TraesCS7A01G560000 | **DHN14** | *DHN14-A1* |
| TraesCS7B01G484900 | | *DHN14-B1* |
| TraesCS7D01G549900 | | *DHN14-D1* |

**Table S15.**

Synteny of wheat dehydrin genes with rice, *Hordeum vulgare*, *Brachypodium distachyon* and *Sorghum bicolor*. Only the HC dehydrins in chromosome A are shown. 'Chr comp': Chromosome compartment as defined by IWGSC (2018). Start pos.: Gene start position at the RefSeqv1 pseudomolecule (IWGSC 2018); *: pseudomolecule percentile

| *Triticum aestivum* | | | | | | *Oryza sativa* | | | *Hordeum vulgare* | | | *Brachypodium distachyon* | | | *Sorghum bicolor* | | |
|---|---|---|---|---|---|---|---|---|---|---|---|---|---|---|---|---|---|
| **A genome gene model** | **Chr. Comp.** | ***** | **DHN name** | **Start pos.** | **Chr** | **Gene** | **Start pos.** | **Chr** | **Gene** | **Start pos.** | **Chr** | **Gene** | **Start pos.** | **Chr** | **Gene** | **Start pos.** | **Chr** |
| TraesCS3A01G254600 | R2B | 63 | *DHN35-A1* | 476564123 | 3A | OS01T0702500-01 | 29123535 | os1 | | | | BRADI2G47580.1 | 47975647 | bd2 | Sb03g032255.1 | 60688846 | sb3 |
| TraesCS3A01G396200 | R2B | 86 | *DHN33-A1* | 643460238 | 3A | | | | HORVU3Hr1G089300.1 | 628030631 | hv3H | | | | | | |
| TraesCS4A01G250900 | R2B | 76 | *DHN6-A1* | 562290028 | 4A | | | | | | | | | | | | |
| TraesCS4A01G299100 | R3 | 80 | *DHN36-A1* | 597754246 | 4A | | | | | | | | | | | | |
| TraesCS5A01G369800 | R3 | 80 | *DHN2-A1* | 569677464 | 5A | | | | HORVU5Hr1G092100.1 | 587585373 | hv5H | | | | | | |
| TraesCS5A01G369900 | R3 | 80 | *DHN1-A1* | 569683002 | 5A | | | | | | | | | | | | |
| TraesCS5A01G424700 | R3 | 86 | *DHN38-A1* | 610078630 | 5A | | | | HORVU5Hr1G103460.2 | 616115076 | hv5H | | | | | | |
| TraesCS5A01G424800 | R3 | 86 | *DHN38-A2* | 610184966 | 5A | | | | | | | | | | | | |
| TraesCS6A01G059800 | R1 | 5 | *DHN37-A1* | 31583758 | 6A | | | | HORVU6Hr1G011050.1 | 19541242 | hv6H | | | | | | |
| TraesCS6A01G253300 | R2B | 76 | *DHN11-A1* | 468474016 | 6A | OS02T0669100-01 | 27165514 | os2 | HORVU6Hr1G064620.1 | 439397806 | hv6H | BRADI3G51200.1 | 52299700 | bd3 | Sb04g032250.1 | 62251614 | sb4 |
| TraesCS6A01G350100 | R3 | 94 | *DHN5-A1* | 581982926 | 6A | | | | | | | | | | | | |
| TraesCS6A01G350200 | R3 | 94 | *DHN9-A1* | 582086404 | 6A | | | | | | | | | | | | |
| TraesCS6A01G350300 | R3 | 94 | *DHN5-A2* | 582092081 | 6A | | | | | | | | | | | | |
| TraesCS6A01G350500 | R3 | 94 | *DHN4-A1* | 582264814 | 6A | | | | HORVU6Hr1G083980.1 | 553165574 | hv6H | | | | | | |
| TraesCS6A01G350600 | R3 | 94 | *DHN3-A1* | 582511184 | 6A | | | | | | | | | | | | |
| TraesCS6A01G350700 | R3 | 94 | *DHN3-A6* | 582516403 | 6A | | | | | | | | | | | | |
| TraesCS6A01G350800 | R3 | 94 | *DHN3-A5* | 582630751 | 6A | | | | | | | | | | | | |
| TraesCS6A01G350900 | R3 | 94 | *DHN3-A7* | 582638141 | 6A | | | | | | | | | | | | |
| TraesCS7A01G560000 | R3 | 99 | *DHN14-A1* | 731882428 | 7A | | | | | | | | | | | | |

**Table S16.**

TFs predicted by genie3 to regulate dehydrins. The top 1 million edges from the genie3 network were used for this analysis (Ramírez-González et al. 2018).

| | Number of TF from each family predicted to regulate | | | Percentage of TF from each family predicted to regulate | | | |
|---|---|---|---|---|---|---|---|
| TF_family | dehydrins | all genes | | dehydrins | all genes | chisq (dehydrins vs all genes) | enriched for dehydrin targets |
| AP2/EREBP | 38 | 352 | | 16.7% | 11.9% | 0.00000 | YES |
| NAC | 31 | 255 | | 13.6% | 8.6% | 0.00000 | YES |
| MYB-related | 29 | 498 | | 12.7% | 16.9% | 0.00000 | NO |
| ABI3/VP1 | 13 | 169 | | 5.7% | 5.7% | 0.95317 | NO |
| HD-Zip_I_II | 13 | 56 | | 5.7% | 1.9% | 0.00000 | YES |
| bHLH | 10 | 207 | | 4.4% | 7.0% | 0.00000 | NO |
| C3H | 10 | 90 | | 4.4% | 3.0% | 0.00002 | YES |
| HSF | 10 | 62 | | 4.4% | 2.1% | 0.00000 | YES |
| WRKY | 9 | 252 | | 3.9% | 8.5% | 0.00000 | NO |
| MADS_II | 8 | 107 | | 3.5% | 3.6% | 0.73360 | NO |
| C2C2_GATA | 5 | 49 | | 2.2% | 1.7% | 0.02358 | YES |
| GRAS | 5 | 86 | | 2.2% | 2.9% | 0.01984 | NO |
| AS2/LOB | 4 | 60 | | 1.8% | 2.0% | 0.28318 | NO |
| C2C2_CO-like | 4 | 27 | | 1.8% | 0.9% | 0.00000 | YES |
| Sigma70-like | 4 | 18 | | 1.8% | 0.6% | 0.00000 | YES |
| TAZ | 4 | 21 | | 1.8% | 0.7% | 0.00000 | YES |
| MADS_I | 3 | 41 | | 1.3% | 1.4% | 0.73277 | NO |
| mTERF | 3 | 85 | | 1.3% | 2.9% | 0.00000 | NO |
| PLATZ | 3 | 32 | | 1.3% | 1.1% | 0.22482 | YES |
| ARF | 2 | 61 | | 0.9% | 2.1% | 0.00001 | NO |
| C2C2_Dof | 2 | 72 | | 0.9% | 2.4% | 0.00000 | NO |
| C2C2_YABBY | 2 | 19 | | 0.9% | 0.6% | 0.11300 | YES |
| CSD | 2 | 15 | | 0.9% | 0.5% | 0.00483 | YES |
| FHA | 2 | 42 | | 0.9% | 1.4% | 0.01227 | NO |
| GARP_G2-like | 2 | 65 | | 0.9% | 2.2% | 0.00000 | NO |
| tify | 2 | 67 | | 0.9% | 2.3% | 0.00000 | NO |
| Zinc finger, MIZ type | 2 | 15 | | 0.9% | 0.5% | 0.00483 | YES |
| BES1 | 1 | 13 | | 0.4% | 0.4% | 0.98736 | NO |
| CPP | 1 | 25 | | 0.4% | 0.8% | 0.01545 | NO |
| E2F/DP | 1 | 22 | | 0.4% | 0.7% | 0.05260 | NO |
| HD_PLINC | 1 | 27 | | 0.4% | 0.9% | 0.00657 | NO |
| SBP | 1 | 35 | | 0.4% | 1.2% | 0.00018 | NO |
| Whirly | 1 | 6 | | 0.4% | 0.2% | 0.00455 | YES |

**Table S17.**

NAC and AP2/EREBP TFs predicted to regulate dehydrins by top 1 million edges from genie3. Homoeologs are indicated in the same color.

| TF | TF family | Arabidopsis orthologue(s) | Rice orthologue(s) | Arabidopsis function | Rice function |
|---|---|---|---|---|---|
| TraesCS1A01G263700 | NAC | AT1G01720.1 | LOC_Os05g34830 | ATAF1 negative regulator of genes in drought stress https://doi.org/1 | OsNAC52, ABA responsive, increases drought tolerance https://doi.org/10.1007/s11240-009-9640-9 |
| TraesCS1D01G194200 | NAC | AT3G49530.1:AT1G54330.1:AT1G33 | LOC_Os10g42130 | NAC062 ABA sensitive doi: 10.4161/psb.11083, mediates protein re-f | unknown |
| TraesCS1D01G263800 | NAC | AT1G01720.1 | LOC_Os05g34830 | ATAF1 negative regulator of genes in drought stress https://doi.org/1 | OsNAC52, ABA responsive, increases drought tolerance https://doi.org/10.1007/s11240-009-9640-9 |
| TraesCS2A01G101400 | NAC | N/A | LOC_Os07g48550 | N/A | ONAC069,ONAC015; pathogen resistance http://agris.fao.org/agris-search/search.do?recordID=KR2012003225 |
| TraesCS2B01G118400 | NAC | N/A | LOC_Os07g48550 | N/A | ONAC069,ONAC015; pathogen resistance http://agris.fao.org/agris-search/search.do?recordID=KR2012003225 |
| TraesCS2B01G118500 | NAC | N/A | LOC_Os07g48550 | N/A | ONAC069,ONAC015; pathogen resistance http://agris.fao.org/agris-search/search.do?recordID=KR2012003225 |
| TraesCS2B01G627200 | NAC | N/A | N/A | N/A | N/A |
| TraesCS2D01G576300 | NAC | N/A | N/A | N/A | N/A |
| TraesCS3B01G271900 | NAC | N/A | LOC_Os01g47670 | N/A | unknown |
| TraesCS3D01G243900 | NAC | N/A | LOC_Os01g47670 | N/A | unknown |
| TraesCS3D01G467000 | NAC | N/A | N/A | N/A | N/A |
| TraesCS4A01G242700 | NAC | AT3G18400.1; AT5G53950.1 | LOC_Os03g42630 | CUC2 leaf shape doi: 10.1105/tpc.106.045617, NAC058 unknown | unknown |
| TraesCS4B01G072400 | NAC | AT5G53950.1; AT3G18400.1 | LOC_Os03g42630 | CUC2 leaf shape doi: 10.1105/tpc.106.045617, NAC058 unknown | unknown |
| TraesCS4D01G071200 | NAC | AT5G53950.1 | LOC_Os03g42630 | CUC2 leaf shape doi: 10.1105/tpc.106.045617 | unknown |
| TraesCS5A01G143200 | NAC | AT3G04070.1 | N/A | NAC047/SHG regulates response to waterlogging doi: 10.1105/tpc.1 | N/A |
| TraesCS5A01G467300 | NAC | N/A | LOC_Os03g59730 | N/A | unknown |
| TraesCS5B01G142100 | NAC | AT3G04070.1 | N/A | NAC047/SHG regulates response to waterlogging doi: 10.1105/tpc.1 | N/A |
| TraesCS5B01G290200 | NAC | N/A | LOC_Os09g33490 | N/A | unknown |
| TraesCS5D01G148800 | NAC | AT3G04070.1 | N/A | NAC047/SHG regulates response to waterlogging doi: 10.1105/tpc.1 | N/A |
| TraesCS7A01G152500 | NAC | N/A | N/A | N/A | N/A |
| TraesCS7A01G209100 | NAC | N/A | LOC_Os12g22940; LOC_O | N/A | ONAC136, unknown |
| TraesCS7A01G375300 | NAC | N/A | N/A | N/A | N/A |
| TraesCS7A01G569100 | NAC | N/A | N/A | N/A | N/A |
| TraesCS7A01G569300 | NAC | N/A | N/A | N/A | N/A |
| TraesCS7B01G004900 | NAC | N/A | N/A | N/A | N/A |
| TraesCS7B01G108900 | NAC | N/A | LOC_Os12g22940; LOC_O | N/A | ONAC136, unknown |
| TraesCS7B01G252100 | NAC | N/A | N/A | N/A | N/A |
| TraesCS7D01G100400 | NAC | N/A | N/A | N/A | N/A |
| TraesCS7D01G154200 | NAC | N/A | N/A | N/A | N/A |
| TraesCS7D01G348100 | NAC | N/A | N/A | N/A | N/A |
| TraesCS7D01G371800 | NAC | N/A | N/A | N/A | N/A |
| TraesCS1A01G244800 | AP2/EREBP | N/A | N/A | N/A | N/A |
| TraesCS1B01G256000 | AP2/EREBP | N/A | N/A | N/A | N/A |
| TraesCS1B01G282300 | AP2/EREBP | N/A | LOC_Os05g36100 | N/A | unknown |
| TraesCS1B01G441300 | AP2/EREBP | N/A | N/A | N/A | N/A |
| TraesCS1D01G244500 | AP2/EREBP | N/A | N/A | N/A | N/A |
| TraesCS2A01G505600 | AP2/EREBP | N/A | LOC_Os04g55970 | N/A | unknown |
| TraesCS2A01G514200 | AP2/EREBP | AT5G67180.1 | LOC_Os04g55560 | N/A | N/A |
| TraesCS2A01G514300 | AP2/EREBP | AT5G67190.1:AT4G36900.1:AT2G23 | LOC_Os04g55520 | TOE3 stem cell nice, DEAR2 DEAR3 CEJ1 closely related to DEAR1 inv | unknown |
| TraesCS2B01G534000 | AP2/EREBP | N/A | LOC_Os04g55970 | N/A | unknown |
| TraesCS2D01G412400 | AP2/EREBP | N/A | LOC_Os04g46400 | N/A | OsDREB4-2 bind dehydration repsonsive element DOI: 10.1111/j.1744-7909.2005.00028.x |
| TraesCS2D01G506500 | AP2/EREBP | N/A | LOC_Os04g55970 | N/A | unknown |
| TraesCS3A01G099200 | AP2/EREBP | N/A | LOC_Os01g07120 | N/A | OSDREB2A improves drought tolerance doi: 10.1016/j.plaphy.2011.09.012 |
| TraesCS3A01G238300 | AP2/EREBP | N/A | LOC_Os01g46870 | N/A | unknown |
| TraesCS3D01G099500 | AP2/EREBP | N/A | N/A | N/A | N/A |
| TraesCS4A01G011600 | AP2/EREBP | N/A | N/A | N/A | N/A |
| TraesCS4A01G123800 | AP2/EREBP | N/A | LOC_Os03g19900 | N/A | unknown |
| TraesCS4B01G180600 | AP2/EREBP | N/A | LOC_Os03g19900 | N/A | unknown |
| TraesCS4B01G292900 | AP2/EREBP | N/A | N/A | N/A | N/A |
| TraesCS4D01G182100 | AP2/EREBP | N/A | LOC_Os03g19900 | N/A | unknown |
| TraesCS4D01G322500 | AP2/EREBP | AT3G25730.1:AT1G13260.1:AT1G25 | N/A | EDF3, RAV1, RAV2,TEM1: RAV1 and RAV2 regulate abiotic stress resp | N/A |
| TraesCS5A01G310700 | AP2/EREBP | N/A | LOC_Os09g35010 | N/A | OsDREB1B confers drought tolerance doi: 10.1007/s11103-008-9391-8 |
| TraesCS5B01G313000 | AP2/EREBP | N/A | LOC_Os09g35030 | N/A | OsDREB1A increases drought tolerance DOI: 10.1046/j.1365-313X.2003.01661.x AND directly induces expression<br>OsDhn1 https://link.springer.com/article/10.1007/s12374-012-0377-3 |
| TraesCS5B01G481300 | AP2/EREBP | N/A | N/A | N/A | N/A |
| TraesCS5D01G317500 | AP2/EREBP | N/A | LOC_Os09g35010 | N/A | OsDREB1B confers drought tolerance doi: 10.1007/s11103-008-9391-8 |
| TraesCS5D01G317600 | AP2/EREBP | N/A | LOC_Os09g35010 | N/A | OsDREB1B confers drought tolerance doi: 10.1007/s11103-008-9391-8 |
| TraesCS5D01G318100 | AP2/EREBP | N/A | LOC_Os09g35020 | N/A | ERF133, unknown |
| TraesCS5D01G318300 | AP2/EREBP | N/A | LOC_Os09g35010 | N/A | OsDREB1B confers drought tolerance doi: 10.1007/s11103-008-9391-8 |
| TraesCS6A01G243500 | AP2/EREBP | N/A | LOC_Os02g43820 | N/A | ERF95 unknown |
| TraesCS6A01G306300 | AP2/EREBP | N/A | N/A | N/A | N/A |
| TraesCS6B01G280800 | AP2/EREBP | N/A | LOC_Os02g43820 | N/A | ERF95 unknown |
| TraesCS6D01G285400 | AP2/EREBP | N/A | N/A | N/A | N/A |
| TraesCS7A01G057800 | AP2/EREBP | N/A | LOC_Os06g03670 | N/A | DREB1C, constitutive expr DOI: 10.1046/j.1365-313X.2003.01661.x |
| TraesCS7B01G074500 | AP2/EREBP | N/A | LOC_Os06g10780 | N/A | unknown |
| TraesCS7D01G052500 | AP2/EREBP | N/A | LOC_Os06g03670 | N/A | DREB1C, constitutive expr DOI: 10.1046/j.1365-313X.2003.01661.x |
| TraesCS7D01G052600 | AP2/EREBP | N/A | LOC_Os06g03670 | N/A | DREB1C, constitutive expr DOI: 10.1046/j.1365-313X.2003.01661.x |
| TraesCS7D01G127600 | AP2/EREBP | AT5G67190.1:AT4G36900.1:AT2G23 | LOC_Os06g07030 | TOE3 stem cell nice, DEAR2 DEAR3 CEJ1 closely related to DEAR1 inv | N/A |
| TraesCS7D01G170600 | AP2/EREBP | N/A | LOC_Os06g10780 | N/A | unknown |
| TraesCS7D01G469200 | AP2/EREBP | N/A | N/A | N/A | N/A |

**Supplementary material File SM1.** SOM and WGCNA modules' gene IDs.
